# Intercept Estimation of Semi‐Parametric Joint Models in the Context of Longitudinal Data Subject to Irregular Observations

**DOI:** 10.1002/bimj.70088

**Published:** 2025-11-06

**Authors:** Luis Ledesma, Eleanor Pullenayegum

**Affiliations:** ^1^ Department of Medicine McMaster University Hamilton Canada; ^2^ Child Health Evaluative Sciences The Hospital for Sick Children Toronto Canada; ^3^ Dalla Lana School of Public Health University of Toronto Toronto Canada

**Keywords:** estimating equations, irregular observation, longitudinal data, semi‐parametric models

## Abstract

Longitudinal data are often subject to irregular visiting times, with outcomes and visit times influenced by a latent variable. Semi‐parametric joint models that account for this dependence have been proposed; among these, the Sun model is the most suitable for count data as it employs a multiplicative link function. Semi‐parametric joint models define an intercept function as the mean outcome when all covariates are set to zero; this is differenced out in the course of estimation and is consequently not estimated. The Sun estimator thus provides estimates of relative covariate effects, but is unable to provide estimates of absolute effects or of longitudinal prognosis in the absence of covariates. We extend the Sun model by additionally estimating the intercept term, showing that our extended estimator is consistent and asymptotically Normal. In simulations, our estimator outperforms the original Sun estimator in terms of bias and standard error and is also more computationally efficient. We apply our estimator to a longitudinal study of tumor recurrence among bladder cancer patients. Provided the intercept term can be adequately captured using splines, we recommend that our extended Sun estimator be used in place of the original estimator, since it leads to smaller bias, smaller standard errors, and allows estimation of the mean outcome trajectories.

## 1 Introduction

Longitudinal studies allow us to study the progression of a disease and risk factors associated with disease progression. However, often measurements are not obtained uniformly through time, with the timing of visits varying across patients; in some scenarios, no two subjects share an observation time. Furthermore, the visit times can be informative, that is, the response variable of interest is related to the visit intensity of a particular subject. For example, visits could be patient‐driven, where a patient being unwell could result in either more visits (due to concerns related to their illness) or fewer visits (due to them being unable to visit the medical center). Failing to account for the times at which measurements are recorded and their relationship with the outcome of interest may result in biased estimation (Buzkova et al. 2010) of the disease process.

Approaches that account for irregularly observed data include methods involving inverse‐intensity weighting and semi‐parametric joint modeling (Pullenayegum and Lim 2016). The main difference between the two methods lies in the assumptions placed on the associations between the outcome and visit time processes. In particular, inverse‐intensity weighting assumes that the outcome and visit time processes are conditionally independent given previously observed outcomes, covariates, and visit times. In contrast, semi‐parametric joint models assume that conditional independence holds given baseline covariates and random effects. Semi‐parametric joint models have been developed for both additive and multiplicative link functions for the conditional mean outcome given covariates and random effects (Pullenayegum and Lim 2016). A multiplicative specification for the outcome model, the Sun model (Sun et al. 2011), is of particular interest when dealing with count data.

Semi‐parametric joint models typically use a nonparametric intercept, where the intercept is the mean outcome as a function of time when all the covariates are set to zero. While the nonparametric formulation of the intercept in semi‐parametric joint models avoids misspecification, it leaves these models unable to estimate disease trajectories over time, and in the case of the Sun model only allows for the interpretation of relative covariate effects. While this could be useful when assessing how the different covariates relate to the outcome of interest, estimating prognosis and absolute effects is often of interest. The Liang additive semi‐parametric joint model (Liang et al. 2009) was recently extended to permit intercept estimation and exhibited smaller standard errors and reduced computation time compared to the original Liang model (Pullenayegum et al. 2023).

In this paper, we propose to extend the Sun semi‐parametric joint model to include the estimation of a parametric functional form for the intercept. We examine the large‐sample properties of this extended estimator and assess its finite sample performance through simulation. Finally, we illustrate the methods developed through application to a randomized trial of tumor recurrence.

## 2 Methods

### 2.1 Notation

For subjects i={1,⋯,n}, let $Y_{i}\left(t\right)$ correspond to a longitudinal outcome at a given time t for t∈[0,τ), where τ is the maximum observation time. Moreover, define $X_{i}$ as a vector of time‐invariant covariates.

Let $N_{i}\left(t\right)$ be a counting process for individual i’s observation times, with associated intensity function λ. Let $C_{i}$ be the censoring time for subject i, and we also define $\Delta_{i}\left(t\right)=I\left(C_{i}\geq t\right)$ as the at‐risk indicator function at time t for subject i.

If β is a parameter of interest in the model, then define $\beta_{0}$ as the true value of the parameter.

### 2.2 The Sun Model

The Sun model considers a multiplicative model for the longitudinal outcome. For a random effect $\nu_{i}$ for subject i with $E(\nu_{i}|X_{i})=1$ (for identifiability), under the Sun model, the outcome and visit intensity models are given by

(1) $$\begin{array}{c} E\left(Y_{i}\left(t\right)|X_{i},\nu_{i}\right)=\nu_{i}\mu_{0}\left(t\right)\exp\left(X_{i}^{\prime }\beta_{0}\right)\\ \lambda \left(t|X_{i},\nu_{i}\right)=\nu_{i}\lambda_{0}\left(t\right)\exp\left(X_{i}^{\prime }\gamma_{0}\right) \end{array},$$

where $\lambda_{0}\left(t\right)$ is a nonparametric baseline intensity function for the intensity model, $\gamma_{0}$ is a regression parameter for the intensity model, $\mu_{0}\left(t\right)$ is a nonparametric intercept term for the outcome model, and $\beta_{0}$, the regression coefficients corresponding to covariates $X_{i}$ for the mean outcome model under a log link, is the parameter of interest. We assume that the outcome and visit processes are conditionally independent given the random effects and covariates.

Estimation of $\beta_{0}$ proceeds as follows. We define a process $M_{i}\left(t\right)$ for arbitrary β and γ by

$$dM_{i}\left(t\right)\left(\beta ,\gamma \right)=\Delta_{i}\left(t\right)Y_{i}\left(t\right)dN_{i}\left(t\right)-\Delta_{i}\left(t\right)\nu_{i}^{2}\lambda_{0}\left(t\right)\mu_{0}\left(t\right)\exp\left(X_{i}^{\prime }\left(\beta +\gamma \right)\right)dt,$$

and note that $dM_{i}\left(t\right)\left(\beta_{0},\gamma_{0}\right)$ is zero mean conditional on $X_{i}$ and $\nu_{i}$. We can thus estimate $\beta_{0}$ by solving the following system of equations:

$$\begin{array}{cc} \sum_{i=1}^{n}dM_{i}\left(t\right)\left(\beta ,\gamma_{0}\right) & =0,\\ \frac{1}{n}\sum_{i=1}^{n}\int_{0}^{\tau }X_{i}dM_{i}\left(t\right)\left(\beta ,\gamma_{0}\right) & =0, \end{array}$$

yielding the following estimating equation for $\hat{\beta }$ (Sun et al. 2011):

(2) $$U\left(\beta ;\gamma \right)=\frac{1}{n}\sum_{i=1}^{n}\int_{0}^{\tau }\left(X_{i}-{\bar{X}}^{*}\left(t;\beta ,\gamma \right)\right)\Delta_{i}\left(t\right)Y_{i}\left(t\right)dN_{i}\left(t\right)=0,$$

where

$${\bar{X}}^{*}\left(t;\beta ,\gamma \right)=\frac{\sum_{i=1}^{n}\Delta_{i}\left(t\right)\nu_{i}^{2}\exp\{X_{i}^{\prime }\left(\beta +\gamma \right)\}X_{i}}{\sum_{i=1}^{n}\Delta_{i}\left(t\right)\nu_{i}^{2}\exp\{X_{i}^{\prime }\left(\beta +\gamma \right)\}}.$$

The random effect $\nu_{i}$ is unobserved, but $\nu_{i}^{2}$ can be estimated through a method of moments by ${\hat{\Omega }}_{i}$, where

$${\hat{\Omega }}_{i}=\frac{m_{i}\left(m_{i}-1\right)}{{\hat{\Lambda }}_{0}{\left(C_{i}\right)}^{2}\exp\left(2X_{i}^{\prime }\hat{\gamma }\right)}$$

for $m_{i}$ being the number of assessments for subject i and $\Lambda_{0}\left(t\right)=\int_{0}^{t}\lambda_{0}\left(u\right)du$. If the censoring is assumed to be non‐informative, we can use estimates ${\hat{\Lambda }}_{0}$ of $\Lambda_{0}$ and $\hat{\gamma }$ of γ from a root‐n consistent estimator $\hat{\gamma }$ of $\gamma_{0}$, such as the Andersen–Gill estimator (Andersen and Gill 1982).

Replacing all these estimates of the intensity model into Equation (2), one derives the following modified estimating equation for $\hat{\beta }$:

$$U\left(\beta ;\hat{\gamma }\right)=\frac{1}{n}\sum_{i=1}^{n}\int_{0}^{\tau }Q\left(t\right)\left(X_{i}-\bar{X}(t;\beta ,\hat{\gamma }\right))\Delta_{i}\left(t\right)Y_{i}\left(t\right)dN_{i}\left(t\right)=0$$

for $\bar{X}$ being ${\bar{X}}^{*}$ but with the estimate ${\hat{\Omega }}_{i}$ of $\nu_{i}^{2}$:

$$\bar{X}\left(t;\beta ,\hat{\gamma }\right)=\frac{\sum_{i=1}^{n}\Delta_{i}\left(t\right){\hat{\Omega }}_{i}\exp\{X_{i}^{\prime }\left(\beta +\hat{\gamma }\right)\}X_{i}}{\sum_{i=1}^{n}\Delta_{i}\left(t\right){\hat{\Omega }}_{i}\exp\{X_{i}^{\prime }\left(\beta +\hat{\gamma }\right)\}}.$$

### 2.3 Estimating the Intercept Term

We now adapt the intercept estimation technique applied in Pullenayegum et al. (2023) to the estimation of the parameters of the Sun multiplicative model. Suppose we model the intercept as $\mu_{0}\left(t\right)=\exp\left(B_{t}{\left(t\right)}^{\prime }\alpha \right)$ for a known vector‐valued function $B_{t}$ of time and parameter α of the same dimension as $B_{t}\left(t\right)$, which is to be estimated. As in Pullenayegum et al. (2023), a spline basis can be used for $B_{t}$ to maintain modeling flexibility.

Let η=(β,α), $\eta_{0}$ be the true value of the parameter, and $Z_{i}\left(t\right)=\left(X_{i},B_{t}\left(t\right)\right)$, and define

$$\begin{array}{cc} dM_{i}^{*}\left(t\right)\left(\eta ,\gamma \right) & =\Delta_{i}\left(t\right)Y_{i}\left(t\right)dN_{i}\left(t\right)-\Delta_{i}\left(t\right)\nu_{i}^{2}\lambda_{0}\left(t\right)\exp(Z_{i}{\left(t\right)}^{\prime }\eta \\ & \;+X_{i}^{\prime }\gamma )dt\\ dM_{i}^{**}\left(t\right)\left(\gamma \right) & =\Delta_{i}\left(t\right)dN_{i}\left(t\right)-\Delta_{i}\left(t\right)\nu_{i}\lambda_{0}\left(t\right)\exp\left(X_{i}^{\prime }\gamma \right)dt. \end{array}$$

Both of these are mean zero for the true values of the parameters; to see that $dM_{i}^{*}\left(t\right)\left(\eta_{0},\gamma_{0}\right)$ is mean zero, notice that $\Delta_{i}\left(t\right),Y_{i}\left(t\right)$, and $N_{i}\left(t\right)$ are conditionally independent given $X_{i}$ and $\nu_{i}$, which allows us to split the expectation of the product into

$$\begin{array}{cc} & E(dM_{i}^{*}\left(t\right)\left(\eta_{0},\gamma_{0}\right)|X_{i},\nu_{i})\\ & =E[\Delta_{i}\left(t\right)Y_{i}\left(t\right)dN_{i}\left(t\right)-\Delta_{i}\left(t\right)\nu_{i}^{2}\lambda_{0}\left(t\right)\exp(Z_{i}{\left(t\right)}^{\prime }\eta_{0}\\ & \;+X_{i}^{\prime }\gamma_{0})dt|X_{i},\nu_{i}]\\ & =E\left(\Delta_{i}\left(t\right)|X_{i},\nu_{i}\right)E\left(Y_{i}\left(t\right)|X_{i},\nu_{i}\right)E\left(dN_{i}\left(t\right)|X_{i},\nu_{i}\right)\\ & \;\;\;\;\;\;\;\;-E\left(\Delta_{i}\left(t\right)|X_{i},\nu_{i}\right)\nu_{i}^{2}\lambda_{0}\left(t\right)\exp\left(Z_{i}{\left(t\right)}^{\prime }\eta_{0}+X_{i}^{\prime }\gamma_{0}\right)dt\\ & =0, \end{array}$$

where we have used Equation (1) for the conditional expectation of the outcome and visit processes. It follows that

(3) $$E\left[\int_{0}^{\tau }Z_{i}\left(t\right)\exp\left(-Z_{i}{\left(t\right)}^{\prime }\eta \right)dM_{i}^{*}\left(t\right)\left(\eta_{0},\gamma_{0}\right)|X_{i},\nu_{i}\right]=0.$$

The motivation behind weighting by $\exp(-Z_{i}{\left(t\right)}^{\prime }\eta )$ is an inverse variance weighting under a Poisson model, which can improve efficiency.

Using the Andersen–Gill estimator $\hat{\gamma }$ of $\gamma_{0}$, by considering Equation (3) and a generalized method of moments approach (Hall 2005), an estimator $\hat{\eta }$ of $\eta_{0}$ can be found as the solution to the system of equations:

(4) $$\begin{array}{cc} \sum_{i=1}^{n}dM_{i}^{**}\left(t\right)\left(\hat{\gamma }\right) & =0, \end{array}$$

(5) $$\begin{array}{cc} \frac{1}{n}\sum_{i=1}^{n}\int_{0}^{\tau }Z_{i}\left(t\right)\exp\left(-Z_{i}{\left(t\right)}^{\prime }\eta \right)dM_{i}^{*}\left(t\right)\left(\eta ,\hat{\gamma }\right) & =0. \end{array}$$

For ease of notation, we will use $dM_{i}^{**}\left(t\right)$ to indicate $dM_{i}^{**}\left(t\right)\left(\hat{\gamma }\right)$ and $dM_{i}^{*}\left(t\right)$ to indicate $dM_{i}^{*}\left(t\right)\left(\eta ,\hat{\gamma }\right)$. Solving Equation (4) yields the usual estimate of the baseline intensity:

$${\hat{\lambda }}_{0}\left(t\right)dt=\frac{\sum_{i=1}^{n}\Delta_{i}\left(t\right)dN_{i}\left(t\right)}{\sum_{i=1}^{n}\Delta_{i}\left(t\right)\nu_{i}\exp\left(X_{i}^{\prime }\hat{\gamma }\right)}.$$

Plugging this into Equation (5), we have

(6) $$\begin{array}{cc} & \sum_{i=1}^{n}\int_{0}^{\tau }Z_{i}\left(t\right)\exp\left(-Z_{i}{\left(t\right)}^{\prime }\eta \right)dM_{i}^{*}\left(t\right)\\ & \;=\sum_{i=1}^{n}\int_{0}^{\tau }Z_{i}\left(t\right)\exp\left(-Z_{i}{\left(t\right)}^{\prime }\eta \right)[\Delta_{i}\left(t\right)Y_{i}\left(t\right)dN_{i}\left(t\right)\\ & \;\;\;\;\;\;\;\;-\Delta_{i}\left(t\right)\nu_{i}^{2}{\hat{\lambda }}_{0}\left(t\right)\exp\left(Z_{i}{\left(t\right)}^{\prime }\eta +X_{i}^{\prime }\hat{\gamma }\right)dt]. \end{array}$$

Now, since $N_{i}$ is a Poisson process,

(7) $$\begin{array}{cc} E(m_{i}|\nu_{i},X_{i},C_{i}) & =\nu_{i}\Lambda_{0}\left(C_{i}\right)\exp\left(X_{i}^{\prime }\gamma_{0}\right),\\ E(m_{i}^{2}|\nu_{i},X_{i},C_{i}) & =\nu_{i}^{2}\Lambda_{0}{\left(C_{i}\right)}^{2}\exp\left(2X_{i}^{\prime }\gamma_{0}\right)+\nu_{i}\Lambda_{0}\left(C_{i}\right)\exp\left(X_{i}^{\prime }\gamma_{0}\right). \end{array}$$

Replacing the expectations with the observed quantities and solving for $\nu_{i}$ and $\nu_{i}^{2}$ using the estimator $\hat{\gamma }$, we propose the method of moments estimators for $\nu_{i}^{2}$ and $\nu_{i}$, ${\hat{\Omega }}_{i}$ and ${\hat{\omega }}_{i}$, respectively:

$$\begin{array}{cc} {\hat{\Omega }}_{i} & =\frac{m_{i}\left(m_{i}-1\right)}{{\hat{\Lambda }}_{0}{\left(C_{i}\right)}^{2}\exp\left(2X_{i}^{\prime }\hat{\gamma }\right)},\\ {\hat{\omega }}_{i} & =\frac{m_{i}}{{\hat{\Lambda }}_{0}\left(C_{i}\right)\exp\left(X_{i}^{\prime }\hat{\gamma }\right)}. \end{array}$$

Plugging in the estimators ${\hat{\lambda }}_{0}\left(t\right)$, $\hat{\Omega_{i}}$, and $\hat{\omega_{i}}$ into Equation (6), one defines:

$$\begin{array}{cc} L(\eta ,\hat{\gamma }) & =\sum_{i=1}^{n}\int_{0}^{\tau }Z_{i}\left(t\right)\exp\left(-Z_{i}{\left(t\right)}^{\prime }\eta \right)\{\Delta_{i}\left(t\right)Y_{i}\left(t\right)dN_{i}\left(t\right)\\ & \;-\Delta_{i}\left(t\right){\hat{\Omega }}_{i}\exp\left(Z_{i}{\left(t\right)}^{\prime }\eta \right)\exp\left(X_{i}^{\prime }\hat{\gamma }\right)\frac{\sum_{i=1}^{n}\Delta_{i}\left(t\right)dN_{i}\left(t\right)}{\sum_{i=1}^{n}\Delta_{i}\left(t\right)\hat{\omega_{i}}\exp\left(X_{i}^{\prime }\hat{\gamma }\right)}\}\\ & =\sum_{i=1}^{n}\int_{0}^{\tau }\left\{Z_{i}\left(t\right)Y_{i}\left(t\right)\exp\left(-Z_{i}{\left(t\right)}^{\prime }\eta \right)\right.\\ & \;-\left.\frac{\sum_{i=1}^{n}\Delta_{i}\left(t\right){\hat{\Omega }}_{i}\exp\left(X_{i}^{\prime }\hat{\gamma }\right)Z_{i}\left(t\right)}{\sum_{i=1}^{n}\Delta_{i}\left(t\right){\hat{\omega }}_{i}\exp\left(X_{i}^{\prime }\hat{\gamma }\right)}\right\}\Delta_{i}\left(t\right)dN_{i}\left(t\right). \end{array}$$

Compared to the original Sun estimation approach, this approach has the benefit that we do not need to estimate η in the inner sum, making estimation less complex.

Letting $L\left(\eta \right)=L\left(\eta ;\hat{\gamma }\right)$, an estimate $\hat{\eta }$ of $\eta_{0}=\left(\beta_{0},\alpha_{0}\right)$ can be obtained by solving L(η)=0, that is,

(8) $$\begin{array}{ccc} & & \sum_{i=1}^{n}\int_{0}^{\tau }\left\{Z_{i}\left(t\right)Y_{i}\left(t\right)\exp\left(-Z_{i}{\left(t\right)}^{\prime }\eta \right)\right.\\ & & \;-\left.\frac{\sum_{i=1}^{n}\Delta_{i}\left(t\right){\hat{\Omega }}_{i}\exp\left(X_{i}^{\prime }\hat{\gamma }\right)Z_{i}\left(t\right)}{\sum_{i=1}^{n}\Delta_{i}\left(t\right){\hat{\omega }}_{i}\exp\left(X_{i}^{\prime }\hat{\gamma }\right)}\right\}\Delta_{i}\left(t\right)dN_{i}\left(t\right)=0, \end{array}$$

which we denote as the extended Sun estimator.

In the Supporting Information, we show that the extended Sun estimator is consistent and asymptotically Normal. We also derive an asymptotic variance estimator; however, we note that, as in Liang et al. (2009), this involves Taylor expansions of complex functions around the infinite‐dimensional parameter $\Lambda_{0}$ and is thus likely to be unstable with small‐ to moderate‐sized samples. Consequently, as in Liang et al. (2009), we recommend the use of the bootstrap to obtain variance estimates.

### 2.4 Goodness‐of‐Fit Diagnostics

In this section, we propose some diagnostics to assess the goodness‐of‐fit for the marginal regression model $E\left(Y_{i}\left(t\right)|X_{i}\right)=Z_{i}\left(t\right)\eta$. Similar to Sun et al. (2011), we define the residual function

$$R_{i}\left(t\right)=\int_{0}^{t}\Delta_{i}\left(u\right)\exp\left(-Z_{i}{\left(u\right)}^{\prime }\hat{\eta }-X_{i}\hat{\gamma }\right)Y_{i}\left(u\right)dN_{i}\left(u\right)-\Omega_{i}{\hat{\Lambda }}_{0}\left(C_{i}\wedge t\right),$$

where ${\hat{\Lambda }}_{0}\left(t\right)$ is the Breslow estimator of the integrated baseline hazard $\Lambda_{0}\left(t\right)=\int_{0}^{u}\lambda_{0}\left(u\right)du$. Sun et al. (2011) discuss assessments for the functional forms of each component of $X_{i}$, and so here we focus on the functional form of the intercept, that is, $B_{t}\left(t\right)$. Since statistical tests often suffer from either over‐powering or under‐powering, we aim to provide diagnostics rather than tests. Let

$$Res\left(t\right)=\frac{1}{n}\sum_{i=1}^{n}R_{i}\left(t\right).$$

In the Supporting Information, we show that Res(t) converges in probability to zero, and we thus suggest plotting Res(t) versus t as a diagnostic for the intercept function.

## 3 Simulation Study

We proceed to study the finite‐sample performance and efficiency of this extended Sun estimator through simulation. Specifically, we seek to (a) verify that the bias and standard errors decrease with increasing sample size and maximum follow‐up time τ; (b) examine how the performance of the estimator varies across different functional forms for the intercept $\mu_{0}\left(t\right)$; (c) examine whether the performance of the estimator varies for binary versus continuous covariates.

We follow a similar simulation setup as in Sun et al. (2011) and Wang et al. (2001). For given $X_{i}$ and $\nu_{i}$, observation times were generated with intensity $\lambda \left(t\right)=\nu_{i}\exp\left(0.5X_{i}\right)$. At each simulated visit time, the response variable was generated as

(9) $$\log Y_{i}\left(t\right)=\log\nu_{i}+\mu_{0}\left(t\right)+X_{i}^{\prime }\beta_{0}+\varepsilon_{i}\left(t\right),$$

where $\varepsilon_{i}\left(t\right)$ is a measurement error generated from an independent N(−0.0625,0.1225) distribution for all t; thus $E(\exp\left(\varepsilon_{i}\left(t\right)\right))=1$, and so we have

$$E\left(Y_{i}\left(t\right)|\nu_{i},X_{i}\right)=\nu_{i}\exp\left(\mu_{0}\left(t\right)\right)\exp\left(X_{i}^{\prime }\beta_{0}\right).$$

The censoring times were taken from the uniform distribution U(1,1.1τ), where this is independent of the covariates and outcome. In addition, administrative censoring was applied at the maximum follow‐up time τ.

Following Sun et al. (2011), when $X_{i}$ was generated from a Bernoulli distribution with expectation $\frac{1}{2}$, the latent variable $\nu_{i}$ was generated by letting

$$\nu_{i}=\exp\left(-\log\left(2.75\right)X_{i}\right)\nu_{i}^{*},$$

where $\nu_{i}^{*}$ is generated from the density function:

$$f\left(\nu_{i}^{*}|X_{i}\right)=\left(1-X_{i}\right)I\left(0.5\leq \nu_{i}^{*}\leq 1.5\right)+\frac{X_{i}}{2.5}I\left(1.5\leq \nu_{i}^{*}\leq 4\right).$$

When $X_{i}$ was generated from a normal distribution N(0,0.25), $\nu_{i}$ is defined as

$$\nu_{i}=\exp\left(-\log\left(2.75\right)I\left(X_{i}\geq 0\right)\right)\nu_{i}^{*}$$

and $\nu_{i}^{*}$ from the density function:

$$f\left(\nu_{i}^{*}|X_{i}\right)=I\left(X_{i}<0\right)I\left(0.5\leq \nu_{i}^{*}\leq 1.5\right)+\frac{I(X_{i}\geq 0)}{2.5}I\left(1.5\leq \nu_{i}^{*}\leq 4\right).$$

In both scenarios, $E(\nu_{i}|X_{i})=1$.

Parameters $\beta_{0}$, n, τ, and $\mu_{0}\left(t\right)$ were varied as shown in Table 1, as was the distribution of $X_{i}$. For each simulation scenario, 505 simulated datasets were generated.

**TABLE 1:** Simulation scenarios: increasing sample size, maximum follow‐up time, varying intercept specification, and changing covariate probability distribution.

Simulation scenario	n	$\beta_{0}$	$\mu_{0}\left(t\right)$	τ	$X_{i}$
1	100,200,250,500	1	log(1+t)+2(t+1)	5	$\mathrm{Ber}(\frac{1}{2})$
2	200	1	log(1+t)+2(t+1)	1.5,3,5	$\mathrm{Ber}(\frac{1}{2})$
3	200	−0.5,0.5,1	log(1+t)+2(t+1)	5	$\mathrm{Ber}(\frac{1}{2})$
			sin(t)+2(t+1)		
			sin(4t)		
4	200	−0.5,0.5,1	log(1+t)+2(t+1)	5	N(0,0.25)
			sin(t)+2(t+1)		
			sin(4t)		

After generating a simulated dataset, we generated a B‐spline basis with degree 3 and 4 knots (at the first to the fourth quintiles) on the simulated visit times. Then, we estimated $\beta_{0}$ using the original Sun model, and $\eta_{0}$ by augmenting the simulated dataset with the B‐spline basis and an intercept term and applying our extended Sun model, and a Generalized Estimating Equation (GEE) (Diggle et al. 2002) with a log link that included the B‐spline basis but ignored the correlation between visit and outcome processes induced by the random effect ν; we refer to this latter model as a GEE with splines model.

The spline basis was fixed across the simulation scenarios to allow for easier interpretability in the results. Estimates were compared in terms of their bias and empirical standard errors (ESE) for the relative effects $\hat{\beta }$. Moreover, we assessed how well the GEE with splines and extended Sun model approaches recover the intercept function term $\mu_{0}\left(t\right)$, by examining the estimates $B{\left(t\right)}^{\prime }\hat{\alpha }$.

In addition, to assess the goodness of fit of the intercept term, we applied our residual diagnostic to one simulated dataset for each choice of $\mu_{0}\left(t\right)$, taking n=200, τ=5, $\beta_{0}=1$, and a binomial distribution of the covariates (see Supporting Information Section B).

### 3.1 Simulation Results

For all sample sizes, the extended Sun model results in a lower bias of the relative effects compared to the other estimation techniques; this bias decreases as sample size increases, as does the ESE (see Table 2). Moreover, the ESE for the extended Sun model is lower than the other two methods for all sample sizes considered. The variance estimates for increasing sample size have also been estimated by using a bootstrap approach (see Table S1).

**TABLE 2:** Bias and ESE estimates for relative effects $\hat{\beta }$, for increasing sample size ($\beta_{0}=1$).

	n=100		n=200		n=350		n=500	
	Bias	ESE	Bias	ESE	Bias	ESE	Bias	ESE
GEE spline model	0.029	0.24	0.035	0.17	0.026	0.13	0.037	0.10
Sun model	0.013	0.52	0.019	0.36	0.049	0.27	0.046	0.23
Extended Sun model	−0.0054	0.11	−0.0068	0.078	−0.0044	0.058	−0.0056	0.049

The smaller bias of the extended Sun estimator over the original Sun and GEE estimator persists as the maximum follow‐up time τ is varied (Table 3). The ESE for the extended Sun model decreases as τ increases, while the ESEs for the other two models increase as τ increases. For the values of τ examined, the extended Sun model had a lower ESE than the regular Sun model.

**TABLE 3:** Bias and ESE estimates for relative effects $\hat{\beta }$, for increasing maximum follow‐up time ($\beta_{0}=1$).

	τ=1.5		τ=3		τ=5	
	Bias	ESE	Bias	ESE	Bias	ESE
GEE spline model	−0.10	0.11	−0.013	0.13	0.035	0.16
Sun model	0.11	0.26	−0.0050	0.29	0.019	0.36
Extended Sun model	0.063	0.18	−0.00062	0.11	−0.0068	0.078

The extended Sun model maintained a smaller bias than the original Sun and GEE models as the intercept was varied, with the exception of the case where $\mu_{0}\left(t\right)=\sin\left(4t\right)$, where the extended Sun and original Sun models had similar biases (see Table 4). For the extended Sun model, the ESE estimates do not appear to greatly change across the different specifications of the intercepts. In contrast, for the other two models, the ESE is reduced for the sinusoidal specification of the intercept. In fact, for this specification of the intercept, the ESE for the extended Sun estimator is higher than that of the GEE with splines and similar to that for the regular Sun estimator. Results were similar when the covariate was Normal rather than Bernoulli.

**TABLE 4:** Bias and ESE estimates for relative effects $\hat{\beta }$, for different intercept terms and $X_{i}$ following different distributions.

	$X_{i}∼\mathrm{Ber}\left(1/2\right)$						$X_{i}∼N\left(0,0.25\right)$					
	$\mu_{0}\left(t\right)=\log\left(1+t\right)+2\left(t+1\right)$		$\mu_{0}\left(t\right)=\sin\left(t\right)+2\left(t+1\right)$		$\mu_{0}\left(t\right)=\sin\left(4t\right)$		$\mu_{0}\left(t\right)=\log\left(1+t\right)+2\left(t+1\right)$		$\mu_{0}\left(t\right)=\sin\left(t\right)+2\left(t+1\right)$		$\mu_{0}\left(t\right)=\sin\left(4t\right)$	
	Bias	ESE	Bias	ESE	Bias	ESE	Bias	ESE	Bias	ESE	Bias	ESE
GEE spline model												
$\beta_{0}=$−0.5	0.039	0.16	0.034	0.11	0.0062	0.060	0.046	0.20	0.022	0.13	0.011	0.065
$\beta_{0}=0.5$	0.024	0.16	0.035	0.11	0.0079	0.057	0.052	0.17	0.038	0.12	−0.0037	0.068
$\beta_{0}=1.0$	0.035	0.16	0.029	0.12	0.013	0.059	0.043	0.19	0.039	0.13	−0.0082	0.074
Sun model												
$\beta_{0}=$−0.5	0.058	0.36	0.023	0.22	−0.0033	0.090	0.085	0.43	0.035	0.27	0.014	0.12
$\beta_{0}=0.5$	0.022	0.35	0.026	0.23	−0.0069	0.090	0.038	0.42	0.014	0.26	−0.018	0.11
$\beta_{0}=1.0$	0.019	0.36	0.014	0.24	−0.0079	0.093	−0.016	0.42	−0.015	0.28	−0.0054	0.12
Extended Sun model												
$\beta_{0}=-0.5$	−0.0053	0.081	−0.0038	0.082	−0.0041	0.085	−0.000010	0.093	−0.0063	0.092	−0.00071	0.10
$\beta_{0}=0.5$	−0.0083	0.080	−0.0028	0.075	−0.0065	0.089	0.00020	0.091	−0.0022	0.085	−0.010	0.10
$\beta_{0}=1.0$	−0.0068	0.078	−0.0054	0.078	−0.0042	0.086	−0.014	0.089	−0.011	0.090	0.0058	0.098

**FIGURE 1:**
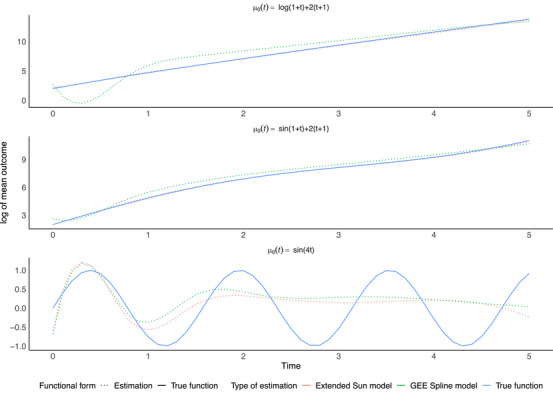
Comparison of the GEE with splines and extended Sun estimation methods for the three specifications of the true intercept function $\mu_{0}\left(t\right)$, with a binary covariate distribution.

For the first two specifications of the intercept term (log‐linear and sine‐linear), the proposed model estimated the outcome trajectory with less bias than the GEE with splines (Figure 1). By contrast, for the sinusoidal intercept, neither model is able to recover the true mean outcome trajectory. Residual diagnostics plots may be found in the Supporting Information. Similar simulation results were observed with a Normal covariate specification.

## 4 Data Analysis

We apply our proposed model to a bladder cancer study conducted by the Veterans Administration Cooperative Urological Research Group (VACURG) (Byar 1980). Patients were randomized to one of three treatment arms: placebo, thiotepa, and pyridoxine; for this analysis, we focus on the placebo (n=47) and thiotepa (n=38) arms. At each visit, the number of new tumors was detected and removed. Other information, such as the number of initial tumors and the size of the largest initial tumor, was also recorded. The maximum follow‐up time in the study is 53 months. For the purposes of analysis, the target of inference in this study is the ratio of the mean number of tumors for the thiotepa versus placebo arms, adjusted for the number of initial tumors.

**FIGURE 2:**
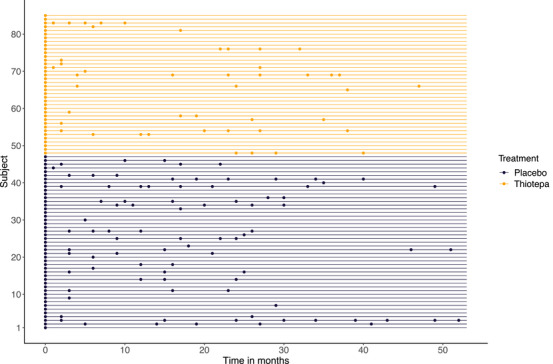
Event times in months for subjects in the veterans' bladder cancer study during the follow‐up period, by treatment arm.

As we can see in Figure 2, visits were irregular. Moreover, subjects who underwent thiotepa treatment had a lower average number of visits, with a mean of 1.18 (standard deviation 1.77) compared to 1.85 (standard deviation 2.25) for those who had a placebo.

We fit both the outcome and visit processes by using Equation (1), using the treatment arm and initial number of tumors as $X_{1},X_{2}$, respectively. Then, the Sun model equations are

$$\begin{array}{c} E\left(Y_{i}\left(t\right)|X_{1},X_{2},\nu_{i}\right)=\nu_{i}\mu_{0}\left(t\right)\exp\left(X_{1}\beta_{1}+X_{2}\beta_{2}\right),\\ \lambda \left(t|X_{1},X_{2},\nu_{i}\right)=\nu_{i}\lambda_{0}\left(t\right)\exp\left(X_{1}\gamma_{1}+X_{2}\gamma_{2}\right). \end{array}$$

The initial number of tumors is centered at the mean to provide interpretability of the mean outcome trajectory, and the placebo is the reference category for treatment. For estimating the visit process, we use an Andersen–Gill model with a frailty effect, using the function coxph() from the package survival. We used a cubic B‐spline with 3 knots for the specification of B(t) by using the function bs() from the package splines to generate the spline basis. For comparison, we also used a GEE with spline basis and the original Sun model. Standard error estimates were computed via a nonparametric bootstrap (Efron and Tibshirani 1994), using 600 resamples.

**FIGURE 3:**
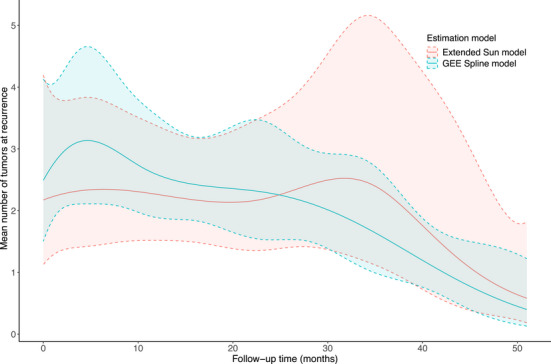
Estimation of the mean number of tumors over time for the veterans' bladder cancer study, along with confidence bands, for both GEE with splines and extended Sun models.

Patients in the thiotepa group had 52% fewer tumors (95% CI 8–75% fewer) compared to patients in the placebo group (see Table 5). The GEE estimated a smaller reduction but with a smaller standard error, while the usual Sun model estimated a larger reduction, but with a larger standard error. The mean outcome trajectories for the number of tumors in the placebo group for both the extended Sun and the GEE models are shown in Figure 3. The residual diagnostic plot suggested no lack of fit of the extended Sun model for our chosen spline basis (see Supporting Information).

**TABLE 5:** Parameter estimates and bootstrap SEs, and 95% confidence intervals for the mean number of tumor ratios, along with point estimates for the veterans' bladder cancer study using different models.

	Parameter estimates (Bootstrap SE)		Mean outcome ratios	
	$\hat{\beta_{1}}$ (SE)	$\hat{\beta_{2}}$ (SE)	Treatment (95% CI)	Initial number of tumors (95% CI)
GEE spline model	−0.66 (0.19)	0.16 (0.045)	0.53 (0.37, 0.77)	1.17 (1.08 1.28)
Sun model	−0.84 (0.39)	0.20 (0.12)	0.45 (0.21, 0.95)	1.20 (1.05, 1.51)
Extended Sun model	−0.72 (0.32)	0.15 (0.067)	0.48 (0.25, 0.90)	1.16 (1.01, 1.32)

## 5 Discussion

In this work, we developed an intercept estimation technique for the multiplicative Sun model for longitudinal data subject to irregular and informative assessment times. We showed that the resulting regression coefficient estimates are consistent and asymptotically Normal. In simulations comparing our approach to the existing Sun model, our approach was able to estimate the outcome trajectory without compromising bias and ESE.

An interesting feature of the simulations was that when the maximum follow‐up time was increased the ESE for both the GEE and the original Sun estimator increased, despite having more data on which to estimate the models. For the GEE with splines model, this is likely due to the informative nature of the visit process not being considered. The increased ESEs for the original Sun estimator could be further examined by testing scenarios where (a) the maximum follow‐up time is fixed, but the rate of visits is increased; (b) the maximum follow‐up time and rate of visits are both increased. This would allow us to disentangle the effects of the number of visits per patient and the duration of follow‐up.

Parameterization of the intercept term through a multidimensional function allows for a spline basis approach in model fitting. In most cases, this performed well in simulations, but resulted in not being able to recover the mean outcome trajectory when the intercept term was sinusoidal. Our recommendation is thus similar to the estimation of another marginal model in the context of informative visits (Coulombe et al. 2021): if the intercept function does not fluctuate extensively through time, then using spline bases provides reasonable estimates; for extensive fluctuation, a nonparametric intercept term may be desirable to avoid misspecification.

While we derived a closed‐form variance estimator, the standard error estimates performed poorly in simulations. Similar behavior has been noted in other semi‐parametric estimation procedures (Liang et al. 2009). As in Liang et al. (2009), we recommend standard error estimates be computed using a nonparametric bootstrap.

The Sun model assumes time‐invariant covariates, and an extension to time dependency could be of interest in certain applications (Sun et al. 2011). Indeed, Equations (1) and (8) can be easily adapted to time‐varying covariates, but one would need to know the value of the covariate process $X_{i}\left(t\right)$ at every point in time, even at points when there are no visits. This limitation was also observed in the original estimation of the Liang model (Liang et al. 2009).

Our proposed estimation procedure allows for the estimation of the outcome trajectory for an outcome measured irregularly over time with observation times linked to the outcome via observed baseline covariates and a random effect. This is helpful in describing prognosis and in quantifying absolute treatment effects. Given that our approach achieves smaller standard errors and is computationally less intensive than the standard Sun model, we suggest that analysts consider using it in place of the standard Sun model when the intercept is not expected to fluctuate drastically over time.

## Author Contributions

EP proposed the topic; LL developed the estimator, conducted the simulations, and analyzed the data with input from EP; LL drafted the manuscript; and EP reviewed the manuscript. Both authors approved the final submitted version.

## Conflicts of Interest

The authors declare no conflicts of interest.

## Open Research Badges

This article has earned an Open Data badge for making publicly available the digitally‐shareable data necessary to reproduce the reported results. The data is available in the Supporting Information section.

This article has earned an open data badge “**Reproducible Research**” for making publicly available the code necessary to reproduce the reported results. The results reported in this article could fully be reproduced.

## Supporting information


**Supporting file 1:** bimj70088‐sup‐0001‐SuppMat.pdf

**Supporting file 2:** bimj70088‐sup‐0002‐DataCode.zip

## Data Availability

The data analyzed in this manuscript are available in the dataset entitled Bladder1 in the survival package on the Comprehensive R Archive Network (CRAN).
