# Supplementary material for: Intercept Estimation of Semi‐Parametric Joint Models in the Context of Longitudinal Data Subject to Irregular Observations
Source: Biom J. 2025 Nov 6;67(6):e70088. doi: 10.1002/bimj.70088 (PMC12592789; doi:10.1002/bimj.70088)
Supplement: Supplementary file 1 — Supporting file 1: bimj70088‐sup‐0001‐SuppMat.pdf [file BIMJ-67-e70088-s001.pdf]

□

APPENDIX

A ASYMPTOTIC PROPERTIES OF THE ESTIMATOR

A.1 Consistency

In a similar manner to Cheng et al [13], we define:

$$D(\eta) = \frac{1}{n}\sum_{i=1}^{n}\int_0^{\tau}\left\{-Y_i(t)\exp(-\mathbf{Z_i}(\mathbf{t})'\eta) - \frac{\sum_{i=1}\Delta_i(t)\hat{\Omega}_i\exp(\mathbf{X_i}'\hat{\gamma})\mathbf{Z_i}(\mathbf{t})\eta}{\sum_{i=1}\Delta_i(t)\hat{\omega}_i\exp(\mathbf{X_i}'\hat{\gamma})}\right\}\Delta_i(t)dN_i(t)$$

notice that $\frac{\partial D(\eta)}{\partial\eta} = \frac{1}{n}L(\eta)$.

By the Strong Law of Large Numbers (SLLN) [14], one has:

$$D(\eta) \xrightarrow{\text{a.s.}} d(\eta) = E\left[\int_0^{\tau}\left[-Y_1(t)e^{-\mathbf{Z_1}(\mathbf{t})'\eta} - \frac{S_1(\eta,t)}{S_0(t)}\right]\Delta_1(t)dN_1(t)\right]$$

where:

$$S_1(\eta,t) = E[\Delta_1(t)\hat{\Omega}_1\exp(\mathbf{X_1}'\hat{\gamma})\mathbf{Z_1}(\mathbf{t})\eta]$$
$$S_0(t) = E[\Delta_1(t)\hat{\omega}_1\exp(\mathbf{X_1}'\hat{\gamma})]$$

Now, the Hessian matrix of D is:

$$\frac{\partial^2 D}{\partial\eta\partial\eta'} = \frac{1}{n}\frac{\partial L}{\partial\eta'} = \frac{1}{n}\sum_{i=1}^{n}\int_0^{\tau}\left[-\mathbf{Z_i}(\mathbf{t})\mathbf{Z_i}(\mathbf{t})'Y_i(t)e^{-\mathbf{Z_i}(\mathbf{t})'\eta}\right]\Delta_i(t)dN_i(t)$$

And, the Hessian of d is:

$$\frac{\partial^2 d}{\partial\eta\partial\eta'} = \int_0^{\tau}E[-\mathbf{Z_1}(\mathbf{t})\mathbf{Z_1}(\mathbf{t})'Y_1(t)e^{-\mathbf{Z_1}(\mathbf{t})'\eta}\Delta_1(t)dN_1(t)]$$

By using the Law of Total Expectation, the expectation in the integrand for $\frac{\partial^2 d}{\partial\eta\partial\eta'}(\eta_0)$ is:

$$\begin{aligned}&E[-\mathbf{Z_1}(\mathbf{t})\mathbf{Z_1}(\mathbf{t})'Y_1(t)e^{-\mathbf{Z_1}(\mathbf{t})'\eta_0}\Delta_1(t)dN_1(t)]\\ &\qquad = E[E\{-\mathbf{Z_1}(\mathbf{t})\mathbf{Z_1}(\mathbf{t})'Y_1(t)e^{-\mathbf{Z_1}(\mathbf{t})'\eta_0}\Delta_1(t)dN_1(t)|\mathbf{X_1},\nu_1,\Delta_1(t)\}]\\ &\qquad = E[-\mathbf{Z_1}(\mathbf{t})\mathbf{Z_1}(\mathbf{t})'e^{-\mathbf{Z_1}(\mathbf{t})\eta_0}\Delta_1(t)E\{Y_1(t)dN_1(t)|\mathbf{X_1},\nu_1,\Delta_1(t)\}]\end{aligned}$$

Notice that, by using conditional independence of the outcome and counting process, conditioned on random effects, covariates, and censoring, along with Equation 1:

$$\begin{aligned}E\{Y_1(t)dN_1(t)|X_1,\nu_1,\Delta_1(t)\} &= E(Y_1(t)|\mathbf{X_1},\nu_1,\Delta_1(t))E(dN_1(t)|\mathbf{X_1},\nu_1,\Delta_1(t))\\ &= \nu_1^2\exp(\mathbf{Z_i}(\mathbf{t})'\eta_0)\lambda_0(t)\exp(\mathbf{X_1}'\gamma_0)dt\end{aligned}$$

Then, if we plug in the above into the integrand term, it follows:

$$\begin{aligned}\frac{\partial^2 d}{\partial\eta\partial\eta'}(\eta_0) &= \int_0^{\tau}E[-\mathbf{Z_1}(\mathbf{t})\mathbf{Z_1}(\mathbf{t})'Y_1(t)e^{-\mathbf{Z_1}(\mathbf{t})'\eta_0}\Delta_1(t)dN_1(t)]\\ &= \int_0^{\tau}E[-\mathbf{Z_1}(\mathbf{t})\mathbf{Z_1}(\mathbf{t})'\nu_1^2\Delta_1(t)e^{\mathbf{X_1}'\gamma_0}]d\Lambda_0(t)\end{aligned}$$

which is a negative definite matrix. By combining this with $d(\eta)$ being a concave function and $\frac{\partial d}{\partial \eta}|_{\eta=\eta_0} = 0$ by the proposed estimating equation, it follows that $d(\eta)$ is uniquely maximized at η_0.

Moreover, $-\frac{\partial^2 D}{\partial\eta\partial\eta'}$ is a positive semi-definite Hessian matrix. It follows that $D(\eta)$ is concave.

Thus $D(\eta)$ converges uniformly in a compact set of η[15], and $\hat{\eta}$ converges to η_0 almost surely as $n \to \infty$[16], which establishes consistency of $\hat{\eta}$.

A.2 Asymptotic normality

Having established the consistency of $\hat{\eta}$, we now show that it is asymptotically normal. Assume that the limit of $\left(\frac{1}{n}\frac{\partial L}{\partial \eta}\right)|_{\eta=\eta_0}$ is a non-singular matrix. Using the fact that $\hat{\eta}$ is consistent and converges to η_0, one expands $L(\eta)$ around η_0 using its Taylor expansion:

$$-\frac{L(\eta_0)}{\sqrt{n}} = \left(\frac{1}{n}\frac{\partial L}{\partial \eta}\right)|_{\eta=\eta_0}\sqrt{n}(\hat{\eta}-\eta_0) + o_p(1)$$

since $\hat{\eta}$ solves $L(\hat{\eta}) = 0$ and where $\left(\frac{1}{n}\frac{\partial L}{\partial \eta}\right)|_{\eta=\eta_0}$ is a constant for large n by the SLLN. Now, one finds:

$$\sqrt{n}(\hat{\eta}-\eta_0) = -\left(\left(\frac{1}{n}\frac{\partial L}{\partial \eta}\right)|_{\eta=\eta_0}\right)^{-1}\frac{L(\eta_0)}{\sqrt{n}} + o_p(1)$$

thus, $\hat{\eta}$ is root-n consistent since the higher ordered terms tend to 0 in probability. It follows that $\sqrt{n}(\hat{\eta}-\eta_0)$ is asymptotically equivalent to:

$$\sqrt{n}(\hat{\eta}-\eta_0) \approx -\left(\left(\frac{1}{n}\frac{\partial L}{\partial \eta}\right)|_{\eta=\eta_0}\right)^{-1}\frac{L(\eta_0)}{\sqrt{n}}$$

By applying the Central Limit Theorem on $\frac{1}{\sqrt{n}}L(\eta_0)$, one finds that $\hat{\eta}$ is asymptotically normal.

A.3 Asymptotic variance

Now, to compute the asymptotic variance, since $\left(\frac{1}{n}\frac{\partial L}{\partial \eta}\right)|_{\eta=\eta_0}$ is a symmetric matrix:

$$\text{var}(\sqrt{n}(\hat{\eta}-\eta_0)) = \left(\left(\frac{1}{n}\frac{\partial L}{\partial \eta}\right)|_{\eta=\eta_0}\right)^{-1}\text{var}\left(\frac{L(\eta_0)}{\sqrt{n}}\right)\left(\left(\frac{1}{n}\frac{\partial L}{\partial \eta}\right)|_{\eta=\eta_0}\right)^{-1}$$

Noting that L is a function of γ, $\omega = (\omega_1, \ldots, \omega_n)$, $\Omega = (\Omega_1, \ldots, \Omega_n)$, we write $L(\eta) = L(\eta; \gamma, \omega, \Omega)$ to make this explicit. Then:

$$\begin{aligned} n^{-1/2}L(\eta_0; \hat{\gamma}, \hat{\omega}, \hat{\Omega}) = n^{-1/2}L(\eta_0; \gamma_0, 1, \sigma^2+1) &+ n^{-1/2}\left[L(\eta_0; \hat{\gamma}, \hat{\omega}, \hat{\Omega}) - L(\eta_0; \gamma_0, \hat{\omega}, \hat{\Omega})\right] \\ &+ n^{-1/2}\left[L(\eta_0; \gamma_0, \hat{\omega}, \hat{\Omega}) - L(\eta_0; \gamma_0, \mathbf{1_n}, \hat{\Omega})\right] \\ &+ n^{-1/2}\left[L(\eta_0; \gamma+0, \mathbf{1_n}, \hat{\Omega}) - L(\eta_0; \gamma_0, \mathbf{1_n}, (\sigma_\nu^2+1)\mathbf{1_n})\right] \end{aligned}$$

where $\mathbf{1_n}$ is an n-dimensional vector of ones.

We have:

$$
\begin{aligned}
& n^{-1/2}\left[L(\eta_0;\hat{\gamma},\hat{\omega},\hat{\Omega}) - L(\eta_0;\gamma_0,\hat{\omega},\hat{\Omega})\right] \\
&= -n^{-1/2}\sum_i \int_0^\tau \frac{\frac{1}{n}\sum_j \Delta_j(t)\hat{\Omega}_j(\exp(X_j\hat{\gamma}) - \exp(X_j\gamma_0))Z_j}{\frac{1}{n}\sum_j \Delta_j(t)\hat{\omega}_j\exp(X_j\hat{\gamma})}\Delta_i(t)dN_i(t) \\
&- n^{-1/2}\sum_i \int_0^\tau \frac{\frac{1}{n}\sum_j \Delta_j(t)\hat{\Omega}_j\exp(X_j\gamma_0)Z_j\left(\frac{1}{n}\sum_j \Delta_j(t)\hat{\omega}_j(\exp(X_j\gamma_0) - \exp(X_j\hat{\gamma}))\right)}{\frac{1}{n}\sum_j \Delta_j(t)\hat{\omega}_j\exp(X_j\hat{\gamma})\frac{1}{n}\sum_j \Delta_j(t)\hat{\omega}_j\exp(X_j\gamma_0)}\Delta_i(t)dN_i(t) \\
&= -n^{-1/2}\sum_i \int_0^\tau \frac{\frac{1}{n}\sum_j \Delta_j(t)\hat{\Omega}_j\exp(X_j\gamma_0)(X_j(\hat{\gamma}-\gamma_0) + o_p(n)))Z_j}{\frac{1}{n}\sum_j \Delta_j(t)\hat{\omega}_j\exp(X_j\hat{\gamma})}\Delta_i(t)dN_i(t) \\
&+ n^{-1/2}\sum_i \int_0^\tau \frac{\frac{1}{n}\sum_j \Delta_j(t)\hat{\Omega}_j\exp(X_j\gamma_0)Z_j\left(\frac{1}{n}\sum_j \Delta_j(t)\hat{\omega}_j\exp(X_j\gamma_0)(X_j(\hat{\gamma}-\gamma_0) + o_p(n))\right)}{\frac{1}{n}\sum_j \Delta_j(t)\hat{\omega}_j\exp(X_j\hat{\gamma})\frac{1}{n}\sum_j \Delta_j(t)\hat{\omega}_j\exp(X_j\gamma_0)}\Delta_i(t)dN_i(t) \\
&= \int_0^\tau \left(-\frac{\frac{1}{n}\sum_j \Delta_j(t)\hat{\Omega}_j\exp(X_j\gamma_0)Z_jX_j}{\frac{1}{n}\sum_j \Delta_j(t)\hat{\omega}_j\exp(X_j\hat{\gamma})} + \frac{\frac{1}{n}\sum_j \Delta_j(t)\hat{\Omega}_j\exp(X_j\gamma_0)Z_j\left(\frac{1}{n}\sum_j \Delta_j(t)\hat{\omega}_j\exp(X_j\gamma_0)X_j\right)}{\frac{1}{n}\sum_j \Delta_j(t)\hat{\omega}_j\exp(X_j\hat{\gamma})\frac{1}{n}\sum_j \Delta_j(t)\hat{\omega}_j\exp(X_j\gamma_0)}\right)\frac{1}{n}\sum_j \Delta_j(t)dN_j(t)n^{1/2}(\hat{\gamma}-\gamma_0) + o_p(n^{1/2})
\end{aligned}
$$

Let

$$
dA(t) = \frac{1}{n}\sum_j \Delta_j(t)dN_j(t)
$$

$$
B(t;\omega,\gamma) = \frac{1}{n}\sum_j \Delta_j(t)\omega_j\exp(X_j\gamma) \qquad BX(t;\omega,\gamma) = \frac{1}{n}\sum_j \Delta_j(t)\omega_j\exp(X_j\gamma)X_j \quad BZX(t;\omega,\gamma) = \frac{1}{n}\sum_j \Delta_j(t)\omega_j\exp(X_j\gamma)Z_jX_j
$$

$$
BZ(t;\omega,\gamma) = \frac{1}{n}\sum_j \Delta_j(t)\omega_j\exp(X_j\gamma)Z_j
$$

$$
S_i(\gamma) = \int_0^\tau (X_i - \bar{X}(t))\Delta_i(t)dN_i(t) \qquad \bar{X}(t) = \frac{\sum_j \Delta_j(t)X_j\exp(X_j\gamma)}{\sum_j \Delta_j(t)\exp(X_j\gamma)} \quad S(\gamma) = \sum_i S_i(\gamma)
$$

It then follows that:

$$
\begin{aligned}
& n^{-1/2}\left[L(\eta_0;\hat{\gamma},\hat{\omega},\hat{\Omega}) - L(\eta_0;\gamma_0,\hat{\omega},\hat{\Omega})\right] \\
&= \int_0^\tau \left(\frac{BZ(t;\hat{\Omega},\gamma_0)BX(t;\hat{\omega},\gamma_0)}{B(t;\hat{\omega},\hat{\gamma})B(t;\hat{\omega},\gamma_0)} - \frac{BZX(t;\hat{\Omega},\gamma_0)}{B(t;\hat{\omega},\hat{\gamma})}\right)dA(t)\left(\frac{1}{n}\frac{\partial S(\gamma)}{\partial\gamma}\mid_{\gamma_0}\right)^{-1} n^{-1/2}\sum_i S_i(\gamma_0) + o_p(n^{1/2})
\end{aligned}
$$

Turning to the next term, we have:

$$
\begin{aligned}
& n^{-1/2}\left[L(\eta_0;\gamma_0,\hat{\omega},\hat{\Omega}) - L(\eta_0;\gamma_0,\mathbf{1_n},\hat{\Omega})\right] \\
&= n^{-1/2}\sum_i \int_0^\tau \Delta_i(t)\exp(X_i\gamma_0)(\hat{\omega}_i - 1)\frac{BZ(t;\hat{\Omega},\gamma_0)dA(t)}{B(t;\hat{\omega},\gamma_0)B(t;\mathbf{1_n},\gamma_0)} \\
&= n^{-1/2}\sum_i \int_0^\tau \Delta_i(t)\exp(X_i\gamma_0)\left(\frac{m_i}{\Lambda_0(C_i)\exp(X_i\gamma_0)}\frac{\Lambda_0(C_i)\exp(X_i\gamma_0)}{\hat{\Lambda}_0(C_i)\exp(X_i\hat{\gamma})} - 1\right)\frac{BZ(t;\hat{\Omega},\gamma_0)dA(t)}{B(t;\hat{\omega},\gamma_0)B(t;\mathbf{1_n},\gamma_0)} \\
&= n^{-1/2}\sum_i \int_0^\tau \Delta_i(t)\exp(X_i\gamma_0)\left(\frac{m_i}{\Lambda_0(C_i)\exp(X_i\gamma_0)}\frac{\Lambda_0(C_i)\exp(X_i(\gamma_0 - \hat{\gamma}))}{\hat{\Lambda}_0(C_i)} - 1\right)\frac{BZ(t;\hat{\Omega},\gamma_0)dA(t)}{B(t;\hat{\omega},\gamma_0)B(t;\mathbf{1_n},\gamma_0)} \\
&= n^{-1/2}\sum_i \int_0^\tau \Delta_i(t)\exp(X_i\gamma_0)\left(\frac{m_i}{\Lambda_0(C_i)\exp(X_i\gamma_0)}\left(1 - X_i(\hat{\gamma}-\gamma_0) + o_p(n)\right)\frac{\Lambda_0(C_i)}{\hat{\Lambda}_0(C_i)} - 1\right)\frac{BZ(t;\hat{\Omega},\gamma_0)dA(t)}{B(t;\hat{\omega},\gamma_0)B(t;\mathbf{1_n},\gamma_0)}
\end{aligned}
$$

Note that:

$$
\begin{aligned}
\hat{\Lambda}_0(C_i) &= \int_0^{C_i} \frac{\frac{1}{n}\sum_j \Delta_j(t)dN_j(t)}{\frac{1}{n}\sum_j \Delta_j(t)\exp(X_j\hat{\gamma})} \\
&= \int_0^{C_i} \frac{\frac{1}{n}\sum_j \Delta_j(t)(dN_j(t) - \exp(X_j\gamma_0)d\Lambda_0(t))}{\frac{1}{n}\sum_j \Delta_j(t)\exp(X_j\hat{\gamma})} + \int_0^{C_i} \frac{\frac{1}{n}\sum_j \Delta_j(t)\exp(X_j\gamma_0)d\Lambda_0(t)}{\frac{1}{n}\sum_j \Delta_j(t)\exp(X_j\hat{\gamma})} \\
&= \int_0^{C_i} \frac{\frac{1}{n}\sum_j \Delta_j(t)dM_j(t)}{\frac{1}{n}\sum_j \Delta_j(t)\exp(X_j\gamma_0)\exp(X_j(\hat{\gamma}-\gamma_0))} + \int_0^{C_i} \frac{\frac{1}{n}\sum_j \Delta_j(t)\exp(X_j\gamma_0)d\Lambda_0(t)}{\frac{1}{n}\sum_j \Delta_j(t)\exp(X_j\gamma_0)\exp(X_j(\hat{\gamma}-\gamma_0))} \\
&= \frac{1}{n^{1/2}}\int_0^{C_i} \frac{\frac{1}{n^{1/2}}\sum_j \Delta_j(t)dM_j(t)}{\frac{1}{n}\sum_j \Delta_j(t)\exp(X_j\gamma_0)(1+o_p(n^{1/2}))} + \int_0^{C_i} \frac{\frac{1}{n}\sum_j \Delta_j(t)\exp(X_j\gamma_0)}{\frac{1}{n}\sum_j \Delta_j(t)\exp(X_j\gamma_0) + \frac{1}{n}\sum_j \Delta_j(t)\exp(X_j\gamma_0)(X_j(\hat{\gamma}-\gamma_0)+o_p(n))}d\Lambda_0(t) \\
&= \frac{1}{n^{1/2}}\int_0^{C_i} \frac{\left(\frac{1}{n^{1/2}}\sum_j \Delta_j(t)dM_j(t)\right)+o_p(n^{1/2})}{\frac{1}{n}\sum_j \Delta_j(t)\exp(X_j\gamma_0)} + \int_0^{C_i} \frac{1}{1+\frac{\frac{1}{n}\sum_j \Delta_j(t)\exp(X_j\gamma_0)(X_j(\hat{\gamma}-\gamma_0)+o_p(n))}{\frac{1}{n}\sum_j \Delta_j(t)\exp(X_j\gamma_0)}}d\Lambda_0(t) \\
&= \frac{1}{n^{1/2}}\int_0^{C_i} \frac{\frac{1}{n^{1/2}}\sum_j \Delta_j(t)dM_j(t)}{\frac{1}{n}\sum_j \Delta_j(t)\exp(X_j\gamma_0)} + \int_0^{C_i}\left(1 - \frac{\frac{1}{n}\sum_j \Delta_j(t)X_j\exp(X_j\gamma_0)(\hat{\gamma}-\gamma_0)}{\frac{1}{n}\sum_j \Delta_j(t)\exp(X_j\gamma_0)}\right)d\Lambda_0(t) + +o_p(n) \\
&= \Lambda_0(C_i) + \frac{1}{n^{1/2}}\int_0^{\tau} \Delta_i(t)\frac{\frac{1}{n^{1/2}}\sum_j \Delta_j(t)dM_j(t)}{\frac{1}{n}\sum_j \Delta_j(t)\exp(X_j\gamma_0)} - \int_0^{\tau} \Delta_i(t)\frac{\frac{1}{n}\sum_j \Delta_j(t)X_j\exp(X_j\gamma_0)(\hat{\gamma}-\gamma_0)}{\frac{1}{n}\sum_j \Delta_j(t)\exp(X_j\gamma_0)}d\Lambda_0(t) + +o_p(n)
\end{aligned}
$$

where $dM_i(t) = dN_i(t) - \exp(X_i\gamma_0)d\Lambda_0(t)$. It follows that:

$$
\begin{aligned}
\frac{\Lambda_0(C_i)}{\hat{\Lambda}_0(C_i)} &= \frac{\Lambda_0(C_i)}{\Lambda_0(C_i) + \frac{1}{n^{1/2}}\int_0^{\tau}\Delta_i(t)\frac{\frac{1}{n^{1/2}}\sum_j \Delta_j(t)dM_j(t)}{\frac{1}{n}\sum_j \Delta_j(t)\exp(X_j\gamma_0)} - \int_0^{\tau}\Delta_i(t)\frac{\frac{1}{n}\sum_j \Delta_j(t)\exp(X_j\gamma_0)X_j(\hat{\gamma}-\gamma_0)}{\frac{1}{n}\sum_j \Delta_j(t)\exp(X_j\gamma_0)}d\Lambda_0(t) + o_p(n)} \\
&= 1 - \frac{1}{\Lambda_0(C_i)}\frac{1}{n^{1/2}}\int_0^{\tau}\Delta_i(t)\frac{\frac{1}{n^{1/2}}\sum_j \Delta_j(t)dM_j(t)}{\frac{1}{n}\sum_j \Delta_j(t)\exp(X_j\gamma_0)} + \frac{1}{\Lambda_0(C_i)}\int_0^{\tau}\Delta_i(t)\frac{\frac{1}{n}\sum_j \Delta_j(t)\exp(X_j\gamma_0)X_j(\hat{\gamma}-\gamma_0)}{\frac{1}{n}\sum_j \Delta_j(t)\exp(X_j\gamma_0)}d\Lambda_0(t) + o_p(n)
\end{aligned}
$$

Consequently,

$$
\begin{aligned}
&n^{-1/2}\left[L(\eta_0;\gamma_0,\hat{\omega},\hat{\Omega})-L(\eta_0;\gamma_0,\mathbf{1_n},\hat{\Omega})\right]\\
&=n^{-1/2}\sum_i\int_0^{\tau}\Delta_i(t)\exp(X_i\gamma_0)\Bigg(\frac{m_i}{\Lambda_0(C_i)\exp(X_i\gamma_0)}\Bigg(1-X_i(\hat{\gamma}-\gamma_0)\\
&\quad-\frac{1}{\Lambda_0(C_i)}\frac{1}{n^{1/2}}\int_0^{\tau}\Delta_i(s)\frac{\frac{1}{n^{1/2}}\sum_j\Delta_j(s)dM_j(s)}{\frac{1}{n}\sum_j\Delta_j(s)\exp(X_j\gamma_0)}+\frac{1}{\Lambda_0(C_i)}\int_0^{\tau}\Delta_i(s)\frac{\frac{1}{n}\sum_j\Delta_j(s)\exp(X_j\gamma_0)X_j(\hat{\gamma}-\gamma_0)}{\frac{1}{n}\sum_j\Delta_j(s)\exp(X_j\gamma_0)}d\Lambda_0(s)\Bigg)-1\Bigg)\frac{BZ(t;\hat{\Omega},\gamma_0)dA(t)}{B(t;\hat{\omega},\gamma_0)B(t;\mathbf{1_n},\gamma_0)}\\
&\quad+o_p(n^{1/2})\\
&=n^{-1/2}\sum_i\int_0^{\tau}\Delta_i(s)\exp(X_i\gamma_0)\Bigg(\frac{m_i}{\Lambda_0(C_i)\exp(X_i\gamma_0)}-1\Bigg)\frac{BZ(t;\hat{\Omega},\gamma_0)dA(t)}{B(t;\hat{\omega},\gamma_0)B(t;\mathbf{1_n},\gamma_0)}\\
&\quad-\int_0^{\tau}\frac{BX(t;\frac{m_i}{\Lambda_0(C_i)\exp(X_j\gamma_0)},\gamma_0)BZ(t;\hat{\Omega},\gamma_0)dA(t)}{B(t;\hat{\omega},\gamma_0)B(t;\mathbf{1_n},\gamma_0)}\left(\frac{1}{n}\frac{\partial S(\gamma)}{\partial\gamma}\mid_{\gamma_0}\right)^{-1}n^{-1/2}\sum_i S_i(\gamma_0)\\
&\quad-\int_0^{\tau}\int_0^{\tau}\frac{\frac{1}{n^{1/2}}\sum_j\Delta_j(s)dM_j(s)}{B(s;1_n,\gamma_0)}\frac{B(t\wedge s;\frac{m_i}{\Lambda_0(C_i)\exp(X_j\gamma_0)},\gamma_0)BZ(t;\hat{\Omega},\gamma_0)dA(t)}{\Lambda_0(C_i)B(t;\hat{\omega},\gamma_0)B(t;\mathbf{1_n},\gamma_0)}\\
&\quad+\int_0^{\tau}\int_0^{\tau}\frac{BX(s;\mathbf{1}_n,\gamma_0)}{B(s;\mathbf{1}_n,\gamma_0)}\frac{B(t\wedge s;\frac{m_i}{\Lambda_0(C_i)\exp(X_j\gamma_0)},\gamma_0)BZ(t;\hat{\Omega},\gamma_0)}{\Lambda_0(C_i)B(t;\hat{\omega},\gamma_0)B(t;\mathbf{1_n},\gamma_0)}dA(t)d\Lambda_0(s)\left(\frac{1}{n}\frac{\partial S(\gamma)}{\partial\gamma}\mid_{\gamma_0}\right)^{-1}n^{-1/2}\sum_i S_i(\gamma_0)+o_p(n^{1/2})\\
&=n^{-1/2}\sum_i\int_0^{\tau}\Delta_i(s)\exp(X_i\gamma_0)\Bigg(\frac{m_i}{\Lambda_0(C_i)\exp(X_i\gamma_0)}-1\Bigg)\frac{BZ(t;\hat{\Omega},\gamma_0)dA(t)}{B(t;\hat{\omega},\gamma_0)B(t;\mathbf{1_n},\gamma_0)}\\
&\quad-\frac{1}{n^{1/2}}\sum_i\int_0^{\tau}\left(\int_0^{\tau}\frac{B(t\wedge s;\frac{m_i}{\Lambda_0(C_i)\exp(X_j\gamma_0)},\gamma_0)BZ(t;\hat{\Omega},\gamma_0)dA(t)}{\Lambda_0(C_i)B(s;\mathbf{1}_n,\gamma_0)B(t;\hat{\omega},\gamma_0)B(t;\mathbf{1_n},\gamma_0)}\right)\Delta_i(s)dM_i(s)\\
&\quad+\int_0^{\tau}\left(\int_0^{\tau}\frac{BX(s;\mathbf{1}_n,\gamma_0)}{B(s;\mathbf{1}_n,\gamma_0)}\frac{B(t\wedge s;\frac{m_i}{\Lambda_0(C_i)\exp(X_j\gamma_0)},\gamma_0)}{\Lambda_0(C_i)}d\Lambda_0(s)-BX(t;\frac{m_i}{\Lambda_0(C_i)\exp(X_j\gamma_0)},\gamma_0)\right)\frac{BZ(t;\hat{\Omega},\gamma_0)dA(t)}{B(t;\mathbf{1_n},\gamma_0)B(t;\hat{\omega},\gamma_0)}\left(\frac{1}{n}\frac{\partial S(\gamma)}{\partial\gamma}\mid_{\gamma_0}\right)^{-1}\\
&\qquad n^{-1/2}\sum_i S_i(\gamma_0)+o_p(n^{1/2})
\end{aligned}
$$

Finally, for the last term, we have:

$$
\begin{aligned}
& n^{-1/2}\left[L(\eta_0;\gamma_0,\mathbf{1_n},\hat{\Omega}) - L(\eta_0;\gamma_0,\mathbf{1_n},(\sigma_\nu^2+1)\mathbf{1_n})\right] \\
&= n^{-1/2}\sum_i\int_0^\tau \frac{\Delta_i(t)\exp(X_i\gamma_0)(\hat{\Omega}_i-(\sigma^2+1))}{B(t;\mathbf{1_n},\gamma_0)}dA(t) \\
&= n^{-1/2}\sum_i\int_0^\tau \frac{\Delta_i(t)\exp(X_i\gamma_0)\left(\frac{m_i(m_i-1)}{\Lambda_0(C_i)^2\exp(2X_i\gamma_0)}\left(\frac{\Lambda_0(C_i)\exp(X_i\gamma_0)}{\hat{\Lambda}_0(C_i)\exp(X_i\hat{\gamma})}\right)^2-(\sigma^2+1)\right)}{B(t;\mathbf{1_n},\gamma_0)}dA(t) \\
&= n^{-1/2}\sum_i\int_0^\tau \frac{\Delta_i(t)\exp(X_i\gamma_0)\left(\frac{m_i(m_i-1)}{\Lambda_0(C_i)^2\exp(2X_i\gamma_0)}\left(\frac{\Lambda_0(C_i)\exp(-X_i(\hat{\gamma}-\gamma_0))}{\hat{\Lambda}_0(C_i)}\right)^2-(\sigma^2+1)\right)}{B(t;\mathbf{1_n},\gamma_0)}dA(t) \\
&= n^{-1/2}\sum_i\int_0^\tau \frac{\Delta_i(t)\exp(X_i\gamma_0)\left(\frac{m_i(m_i-1)}{\Lambda_0(C_i)^2\exp(2X_i\gamma_0)}\left(\frac{\Lambda_0(C_i)}{\hat{\Lambda}_0(C_i)}\right)^2(1-2X_i(\hat{\gamma}-\gamma_0)+o_p(n))-(\sigma^2+1)\right)}{B(t;\mathbf{1_n},\gamma_0)}dA(t) \\
&= n^{-1/2}\sum_i\int_0^\tau \frac{\Delta_i(t)\exp(X_i\gamma_0)\left(\frac{m_i(m_i-1)}{\Lambda_0(C_i)^2\exp(2X_i\gamma_0)}\left(\frac{\Lambda_0(C_i)}{\hat{\Lambda}_0(C_i)}\right)^2(1-2X_i(\hat{\gamma}-\gamma_0))-(\sigma^2+1)\right)}{B(t;\mathbf{1_n},\gamma_0)}dA(t)+o_p(n^{1/2}) \\
&= n^{-1/2}\sum_i\int_0^\tau \Delta_i(t)\exp(X_i\gamma_0)\Bigg(\frac{m_i(m_i-1)}{\Lambda_0(C_i)^2\exp(2X_i\gamma_0)}\Bigg(\\
&\quad 1-\frac{2}{\Lambda_0(C_i)}\frac{1}{n^{1/2}}\int_0^\tau \Delta_i(s)\frac{\frac{1}{n^{1/2}}\sum_j\Delta_j(s)dM_j(s)}{\frac{1}{n}\sum_j\Delta_j(s)\exp(X_j\gamma_0)}+\frac{2}{\Lambda_0(C_i)}\int_0^\tau \Delta_i(s)\frac{\frac{1}{n}\sum_j\Delta_j(s)\exp(X_j\gamma_0)X_j(\hat{\gamma}-\gamma_0)}{\frac{1}{n}\sum_j\Delta_j(t)\exp(X_j\gamma_0)}d\Lambda_0(t)\Bigg) \\
&\quad (1-2X_i(\hat{\gamma}-\gamma_0))-(\sigma^2+1)\Bigg)\frac{dA(t)}{B(t;\mathbf{1_n},\gamma_0)}+o_p(n^{1/2}) \\
&= n^{-1/2}\sum_i\int_0^\tau \Delta_i(t)\exp(X_i\gamma_0)\left(\frac{m_i(m_i-(\sigma^2+1))}{\Lambda_0(C_i)^2\exp(2X_i\gamma_0)}-1\right)\frac{dA(t)}{B(t;\mathbf{1_n},\gamma_0)} \\
&\quad -2\int_0^\tau\int_0^\tau \frac{1}{n}\sum_j\frac{\Delta_j(t\wedge s)m_j(m_j-1)}{\Lambda_0(C_j)^3\exp(X_j\gamma_0)}\frac{\frac{1}{n^{1/2}}\sum_j\Delta_j(s)dA(t)dM_j(s)}{B(s;\mathbf{1}_n,\gamma_0)B(t;\mathbf{1_n},\gamma_0)} \\
&\quad +2\left(\int_0^\tau\int_0^\tau \frac{1}{n}\sum_j\frac{\Delta_j(t\wedge s)m_j(m_j-1)}{\Lambda_0(C_i)^3\exp(X_i\gamma_0)}\frac{BX(s,\mathbf{1_n},\gamma_0)}{B(s;\mathbf{1}_n,\gamma_0)}\frac{d\Lambda_0(s)dA(t)}{B(t;\mathbf{1}_n,\gamma_0)}\right)\left(\frac{1}{n}\frac{\partial S_\gamma}{\partial\gamma}\mid_{\gamma=\gamma_0}\right)\frac{1}{n^{1/2}}\sum_i S_{\gamma_i} \\
&\quad -\int_0^\tau BX\left(t;\frac{m_i(m_i-1)}{\Lambda_0(C_i)^2\exp(2X_i\gamma_0)},\gamma_0\right)\frac{dA(t)}{B(t;\mathbf{1_n},\gamma_0)}\left(\frac{1}{n}\frac{\partial S_\gamma}{\partial\gamma}\mid_{\gamma=\gamma_0}\right)\frac{1}{n^{1/2}}\sum_i S_{\gamma_i}+o_p(n^{1/2}) \\
&= n^{-1/2}\sum_i\int_0^\tau \Delta_i(t)\exp(X_i\gamma_0)\left(\frac{m_i(m_i-(\sigma^2+1))}{\Lambda_0(C_i)^2\exp(2X_i\gamma_0)}-1\right)\frac{dA(t)}{B(t;\mathbf{1_n},\gamma_0)} \\
&\quad -2n^{-1/2}\sum_i\int_0^\tau \Delta_i(s)dM_i(s)\int_0^\tau\frac{1}{n}\sum_j\frac{\Delta_j(t\wedge s)m_j(m_j-1)}{\Lambda_0(C_j)^3\exp(X_j\gamma_0)}\frac{dA(t)}{B(s;\mathbf{1}_n,\gamma_0)B(t;\mathbf{1_n},\gamma_0)} \\
&\quad +2\left(\int_0^\tau\left(\int_0^\tau\frac{1}{n}\sum_j\frac{\Delta_j(t\wedge s)m_j(m_j-1)}{\Lambda_0(C_i)^3\exp(X_i\gamma_0)}\frac{BX(s,\mathbf{1_n},\gamma_0)}{B(s;\mathbf{1}_n,\gamma_0)}d\Lambda_0(s)\right)-BX\left(t;\frac{m_i(m_i-1)}{\Lambda_0(C_i)^2\exp(2X_i\gamma_0)},\gamma_0\right)\right)\frac{dA(t)}{B(t;\mathbf{1_n},\gamma_0)} \\
&\quad \left(\frac{1}{n}\frac{\partial S_\gamma}{\partial\gamma}\mid_{\gamma=\gamma_0}\right)\frac{1}{n^{1/2}}\sum_i S_{\gamma_i}+o_p(n^{1/2})
\end{aligned}
$$

Putting these terms together, we have:

$$
\begin{aligned}
&n^{-1/2}L(\eta_0;\hat{\gamma},\hat{\omega},\hat{\Omega})\\
&= n^{-1/2}L(\eta_0;\gamma_0,1,\sigma^2+1)\\
&+\int_0^{\tau}\left(\frac{BZ(t;\hat{\Omega},\gamma_0)BX(t;\hat{\omega},\gamma_0)}{B(t;\hat{\omega},\hat{\gamma})B(t;\hat{\omega},\gamma_0)}-\frac{BZX(t;\hat{\Omega},\gamma_0)}{B(t;\hat{\omega},\hat{\gamma})}\right)dA(t)\left(\frac{1}{n}\frac{\partial S(\gamma)}{\partial\gamma}\mid_{\gamma_0}\right)^{-1}n^{-1/2}\sum_i S_i(\gamma_0)\\
&+n^{-1/2}\sum_i\int_0^{\tau}\Delta_i(s)\exp(X_i\gamma_0)\left(\frac{m_i}{\Lambda_0(C_i)\exp(X_i\gamma_0)}-1\right)\frac{BZ(t;\hat{\Omega},\gamma_0)dA(t)}{B(t;\hat{\omega},\gamma_0)B(t;\mathbf{1_n},\gamma_0)}\\
&-\frac{1}{n^{1/2}}\sum_i\int_0^{\tau}\left(\int_0^{\tau}\frac{B(t\wedge s;\frac{m_i}{\Lambda_0(C_i)\exp(X_j\gamma_0)},\gamma_0)BZ(t;\hat{\Omega},\gamma_0)dA(t)}{\Lambda_0(C_i)B(s;\mathbf{1}_n,\gamma_0)B(t;\hat{\omega},\gamma_0)B(t;\mathbf{1_n},\gamma_0)}\right)\Delta_i(s)dM_i(s)\\
&+\int_0^{\tau}\left(\int_0^{\tau}\frac{BX(s;\mathbf{1}_n,\gamma_0)}{B(s;\mathbf{1}_n,\gamma_0)}\frac{B(t\wedge s;\frac{m_i}{\Lambda_0(C_i)\exp(X_j\gamma_0)},\gamma_0)}{\Lambda_0(C_i)}d\Lambda_0(s)-BX(t;\frac{m_i}{\Lambda_0(C_i)\exp(X_j\gamma_0)},\gamma_0)\right)\frac{BZ(t;\hat{\Omega},\gamma_0)dA(t)}{B(t;\mathbf{1_n},\gamma_0)B(t;\hat{\omega},\gamma_0)}\left(\frac{1}{n}\frac{\partial S(\gamma)}{\partial\gamma}\mid_{\gamma_0}\right)^{-1}\\
&\qquad n^{-1/2}\sum_i S_i(\gamma_0)\\
&+n^{-1/2}\sum_i\int_0^{\tau}\Delta_i(t)\exp(X_i\gamma_0)\left(\frac{m_i(m_i-1)}{\Lambda_0(C_i)^2\exp(2X_i\gamma_0)}-(\sigma^2+1)\right)\frac{dA(t)}{B(t;\mathbf{1_n},\gamma_0)}\\
&-2n^{-1/2}\sum_i\int_0^{\tau}\Delta_i(s)dM_i(s)\int_0^{\tau}\frac{1}{n}\sum_j\frac{\Delta_j(t\wedge s)m_j(m_j-1)}{\Lambda_0(C_j)^3\exp(X_j\gamma_0)}\frac{dA(t)}{B(s;\mathbf{1}_n,\gamma_0)B(t;\mathbf{1_n},\gamma_0)}\\
&+2\left(\int_0^{\tau}\left(\int_0^{\tau}\frac{1}{n}\sum_j\frac{\Delta_j(t\wedge s)m_j(m_j-1)}{\Lambda_0(C_i)^3\exp(X_i\gamma_0)}\frac{BX(s,\mathbf{1_n},\gamma_0)}{B(s;\mathbf{1}_n,\gamma_0)}d\Lambda_0(s)\right)-BX\left(t;\frac{m_i(m_i-1)}{\Lambda_0(C_i)^2\exp(2X_i\gamma_0)},\gamma_0\right)\right)\frac{dA(t)}{B(t;\mathbf{1_n},\gamma_0)}\\
&\left(\frac{1}{n}\frac{\partial S(\gamma)}{\partial\gamma}\mid_{\gamma=\gamma_0}\right)\frac{1}{n^{1/2}}\sum_i S_i(\gamma_0)+o_p(n^{1/2}).
\end{aligned}
$$

Define:

$$
\begin{aligned}
C_\gamma&=\int_0^{\tau}\left(\frac{BZ(t;\hat{\Omega},\gamma_0)BX(t;\hat{\omega},\gamma_0)}{B(t;\hat{\omega},\hat{\gamma})B(t;\hat{\omega},\gamma_0)}-\frac{BZX(t;\hat{\Omega},\gamma_0)}{B(t;\hat{\omega},\hat{\gamma})}\right)dA(t)\\
&+\int_0^{\tau}\left(\int_0^{\tau}\frac{BX(s;\mathbf{1}_n,\gamma_0)}{B(s;\mathbf{1}_n,\gamma_0)}\frac{B(t\wedge s;\frac{m_i}{\Lambda_0(C_i)\exp(X_j\gamma_0)},\gamma_0)}{\Lambda_0(C_i)}d\Lambda_0(s)-BX(t;\frac{m_i}{\Lambda_0(C_i)\exp(X_j\gamma_0)},\gamma_0)\right)\frac{BZ(t;\hat{\Omega},\gamma_0)dA(t)}{B(t;\mathbf{1_n},\gamma_0)B(t;\hat{\omega},\gamma_0)}\\
&2\left(\int_0^{\tau}\left(\int_0^{\tau}\frac{1}{n}\sum_j\frac{\Delta_j(t\wedge s)m_j(m_j-1)}{\Lambda_0(C_i)^3\exp(X_i\gamma_0)}\frac{BX(s,\mathbf{1_n},\gamma_0)}{B(s;\mathbf{1}_n,\gamma_0)}d\Lambda_0(s)\right)-BX\left(t;\frac{m_i(m_i-1)}{\Lambda_0(C_i)^2\exp(2X_i\gamma_0)},\gamma_0\right)\right)\frac{dA(t)}{B(t;\mathbf{1_n},\gamma_0)}\\
C_M(t)&=\frac{1}{B(t;\mathbf{1_n},\gamma_0)}\int_0^{\tau}\left(\frac{2}{n}\sum_j\frac{\Delta_j(t\wedge s)m_j(m_j-1)}{\Lambda_0(C_j)^3\exp(X_j\gamma_0)}+\frac{B(t\wedge s;\frac{m_i}{\Lambda_0(C_i)\exp(X_j\gamma_0)},\gamma_0)BZ(s;\hat{\Omega},\gamma_0)}{B(s;\hat{\omega},\gamma_0)}\right)\frac{dA(s)}{B(s;\mathbf{1}_n,\gamma_0)}\\
\phi_i&=C_\gamma\left(\frac{1}{n}\frac{\partial S(\gamma_0)}{\partial\gamma}\mid_{\gamma=\gamma_0}\right)S_i(\gamma_0)-\int_0^{\tau}C_M(t)\Delta_i(t)dM_i(t)+\int_0^{\tau}\Delta_i(s)\exp(X_i\gamma_0)\left(\frac{m_i}{\Lambda_0(C_i)\exp(X_i\gamma_0)}-1\right)\frac{BZ(t;\hat{\Omega},\gamma_0)dA(t)}{B(t;\hat{\omega},\gamma_0)B(t;\mathbf{1_n},\gamma_0)}\\
&+\int_0^{\tau}\Delta_i(t)\exp(X_i\gamma_0)\left(\frac{m_i(m_i-1)}{\Lambda_0(C_i)^2\exp(2X_i\gamma_0)}-(\sigma^2+1)\right)\frac{dA(t)}{B(t;\mathbf{1_n},\gamma_0)}
\end{aligned}
$$

Note that

$$
0=L(\hat{\eta};\hat{\gamma},\omega,\Omega)=L(\eta_0;\hat{\gamma},\omega,\Omega)+\left(\frac{\partial L}{\partial\eta}\mid_{\eta=\eta_0}\right)(\hat{\eta}-\eta_0)+o_p(n^{1/2}),
$$

and so

$$
\begin{aligned}
n^{1/2}(\hat{\eta}-\eta_0) &= \left(\frac{1}{n}\frac{\partial L}{\partial \eta}\mid_{\eta=\eta_0}\right)^{-1} n^{-1/2}L(\eta_0;\hat{\gamma},\omega,\Omega)+o_p(1)\\
&= \left(\frac{1}{n}\frac{\partial L}{\partial \eta}\mid_{\eta=\eta_0}\right)^{-1} n^{-1/2}\left[L(\eta_0;\gamma_0,\mathbf{1}_n,\mathbf{1_n}(\sigma^2+1))+\sum_i \phi_i\right]+o_p(1)\\
&= \left(\frac{1}{n}\frac{\partial L}{\partial \eta}\mid_{\eta=\eta_0}\right)^{-1} n^{-1/2}\sum_i\left[\int_0^\tau\left(Z_i(t)Y_i(t)exp(Z_i(t)\eta_0)-\frac{BZ(t;\gamma_0,\mathbf{1_n}(\sigma^2+1))}{B(t;\gamma_0,\mathbf{1_n})}\right)\Delta_i(t)dN_i(t)+\phi_i\right]+o_p(1)
\end{aligned}
$$

It follows that

$$
var(\hat{\eta})=\left(\frac{1}{n}\frac{\partial L}{\partial \eta}\mid_{\eta=\eta_0}\right)^{-1}\frac{1}{n}var\left(\int_0^\tau\left(Z_i(t)Y_i(t)exp(Z_i(t)\eta_0)-\frac{BZ(t;\gamma_0,\mathbf{1_n}(\sigma^2+1))}{B(t;\gamma_0,\mathbf{1_n})}\right)\Delta_i(t)dN_i(t)+\phi_i\right)\left(\frac{1}{n}\frac{\partial L}{\partial \eta'}\mid_{\eta=\eta_0}\right)^{-1}
$$

A.4 Diagnostics

In this section we show that the residual function $Res(t)$ is asymptotically Normal with mean 0.

First, we write $Res(t)=Res(t;\eta,\gamma,\Lambda_0,\Omega)$ to make the dependence on these parameters explicit. Next, note that:

$$
Res(t;\hat{\eta},\hat{\gamma},\hat{\Lambda}_0,\hat{\Omega})=Res(t;\eta_0,\gamma_0,\Lambda_0,\nu^2)+\left(Res(t;\hat{\eta},\hat{\gamma}_0,\hat{\Lambda}_0,\Omega)-Res(t;\eta_0,\gamma_0,\Lambda_0,\nu^2)\right).
$$

We have that

$$
n^{1/2}Res(t;\eta_0,\gamma_0,\Lambda_0,\nu^2)=R_i(t)=\frac{1}{\sqrt{n}}\sum_{i=1}^n\left(\int_0^t\Delta_i(u)\exp(-\mathbf{Z}_i(u)'\eta_0-\mathbf{X}_i\gamma_0)Y_i(u)dN_i(u)-\nu_i^2\Lambda_0(C_i\wedge t).\right)
$$

Since each component of the sum is independent of the others and zero mean, it follows that $n^{1/2}Res(t;\eta_0,\gamma_0,\Lambda_0,\nu^2)$ is asymptotically Normal with mean zero.

Turning now to the second term, note that:

$$
\begin{aligned}
&n^{1/2}\left(Res(t;\hat{\eta},\hat{\gamma},\hat{\Lambda}_0,\Omega)-Res(t;\eta_0,\gamma_0,\Lambda_0,\nu^2)\right)\\
&=n^{1/2}\left(Res(t;\hat{\eta},\hat{\gamma},\Lambda_0,\nu_i)-Res(t;\eta_0,\gamma_0,\Lambda_0,\nu^2)\right)+n^{1/2}\left(Res(t;\eta_0,\gamma_0,\hat{\Lambda}_0,\Omega)-Res(t;\eta_0,\gamma_0,\Lambda_0,\nu^2)\right)\\
&=\frac{1}{\sqrt{n}}\sum_{i=1}^n\int_0^t\Delta_i(u)\exp(-\mathbf{Z}_i(u)'\eta_0-\mathbf{X}_i\gamma_0)Y_i(u)\left(\exp(-(Z_i(u)(\hat{\eta}-\eta_0)+X_i(\hat{\gamma}-\gamma_0)-1)\right)dN_i(u)\\
&\quad-\frac{1}{\sqrt{n}}\sum_{i=1}^n\left((\hat{\Omega}_i-\nu_i^2)\hat{\Lambda}_0(C_i\wedge t)-\nu_i^2(\hat{\Lambda}_0(C_i\wedge t)-\Lambda_0(C_i\wedge t))\right)\\
&=\frac{1}{\sqrt{n}}\sum_{i=1}^n\int_0^t\Delta_i(u)\exp(-\mathbf{Z}_i(u)'\eta_0-\mathbf{X}_i\gamma_0)Y_i(u)\left(-Z_i(u)(\hat{\eta}-\eta_0)-X_i(\hat{\gamma}-\gamma_0)+o_p(n^{1/2})\right)dN_i(u)\\
&\quad-\frac{1}{\sqrt{n}}\sum_{i=1}^n\left(\left(\frac{m_i(m_i-1)}{\hat{\Lambda}_0(C_i)^2\exp(2X_i\hat{\gamma})}-\nu_i^2\right)\hat{\Lambda}_0(C_i\wedge t)-\nu_i^2(\hat{\Lambda}_0(C_i\wedge t)-\Lambda_0(C_i\wedge t))\right)\\
&=-\frac{1}{n}\sum_{i=1}^n\int_0^t\Delta_i(u)\exp(-\mathbf{Z}_i(u)'\eta_0-\mathbf{X}_i\gamma_0)Y_i(u)Z_i(u)dN_i(u)n^{1/2}(\hat{\eta}-\eta_0)\\
&\quad-\frac{1}{n}\sum_{i=1}^n\int_0^t\Delta_i(u)\exp(-\mathbf{Z}_i(u)'\eta_0-\mathbf{X}_i\gamma_0)Y_i(u)X_idN_i(u)n^{1/2}(\hat{\gamma}-\gamma_0)\\
&\quad-\frac{1}{\sqrt{n}}\sum_{i=1}^n\left(\left(\frac{m_i(m_i-1)}{\Lambda_0(C_i)^2\exp(2X_i\gamma_0)}\left(\frac{\Lambda_0(C_i)\exp(X_i\gamma_0)}{\hat{\Lambda}_0(C_i)\exp(X_i\hat{\gamma})}\right)^2-\nu_i^2\right)\hat{\Lambda}_0(C_i\wedge t)-\nu_i^2(\hat{\Lambda}_0(C_i\wedge t)-\Lambda_0(C_i\wedge t))\right)+o_p(1)
\end{aligned}
$$

As noted above,

$$\begin{aligned}\frac{\Lambda_0(C_i)}{\hat{\Lambda}_0(C_i)} &= 1 - \frac{1}{\Lambda_0(C_i)}\frac{1}{n^{1/2}}\int_0^\tau \Delta_i(t)\frac{\frac{1}{n^{1/2}}\sum_j \Delta_j(t)dM_j(t)}{\frac{1}{n}\sum_j \Delta_j(t)\exp(X_j\gamma_0)} + \frac{1}{\Lambda_0(C_i)}\int_0^\tau \Delta_i(t)\frac{\frac{1}{n}\sum_j \Delta_j(t)\exp(X_j\gamma_0)X_j(\hat{\gamma}-\gamma_0)}{\frac{1}{n}\sum_j \Delta_j(t)\exp(X_j\gamma_0)}d\Lambda_0(t) + o_p(n)\\ &= 1 + o_p(1)\end{aligned}$$

Furthermore, $n^{1/2}(\hat{\Lambda}_0(t) - \Lambda_0(t))$ converges in distribution to a Normal distribution with mean 0. It follows that

$$\begin{aligned}&\frac{1}{\sqrt{n}}\sum_{i=1}^n \left(\frac{m_i(m_i-1)}{\Lambda_0(C_i)^2\exp(2X_i\gamma_0)}\left(\frac{\Lambda_0(C_i)\exp(X_i\gamma_0)}{\hat{\Lambda}_0(C_i)\exp(X_i\hat{\gamma})}\right)^2 - \nu_i^2\right)\hat{\Lambda}_0(C_i\wedge t)\\
&= \frac{1}{\sqrt{n}}\sum_{i=1}^n \left(\frac{m_i(m_i-1)}{\Lambda_0(C_i)^2\exp(2X_i\gamma_0)} - \nu_i^2\right)\hat{\Lambda}_0(C_i\wedge t)\\
&\quad - \frac{2}{n}\sum_{i=1}^n \frac{m_i(m_i-1)}{\Lambda_0(C_i)^2\exp(2X_i\gamma_0)}\frac{\hat{\Lambda}_0(C_i\wedge t)}{\Lambda_0(C_i)}\int_0^\tau \Delta_i(t)\frac{\frac{1}{n^{1/2}}\sum_j \Delta_j(t)dM_j(t)}{\frac{1}{n}\sum_j \Delta_j(t)\exp(X_j\gamma_0)}\\
&\quad + \frac{2}{n}\sum_{i=1}^n \frac{m_i(m_i-1)}{\Lambda_0(C_i)^2\exp(2X_i\gamma_0)}\frac{\hat{\Lambda}_0(C_i\wedge t)}{\Lambda_0(C_i)}\int_0^\tau \Delta_i(t)\frac{\frac{1}{n}\sum_j \Delta_j(t)\exp(X_j\gamma_0)X_j}{\frac{1}{n}\sum_j \Delta_j(t)\exp(X_j\gamma_0)}d\Lambda_0(t)n^{1/2}(\hat{\gamma}-\gamma_0) + o_p(n^{1/2})\\
&= \frac{1}{\sqrt{n}}\sum_{i=1}^n \left(\frac{m_i(m_i-1)}{\Lambda_0(C_i)^2\exp(2X_i\gamma_0)} - \nu_i^2\right)\Lambda_0(C_i\wedge t)\\
&\quad - \frac{2}{n}\sum_{i=1}^n \frac{m_i(m_i-1)}{\Lambda_0(C_i)^2\exp(2X_i\gamma_0)}\frac{\Lambda_0(C_i\wedge t)}{\Lambda_0(C_i)}\int_0^\tau \Delta_i(t)\frac{\frac{1}{n^{1/2}}\sum_j \Delta_j(t)dM_j(t)}{\frac{1}{n}\sum_j \Delta_j(t)\exp(X_j\gamma_0)}\\
&\quad + \frac{2}{n}\sum_{i=1}^n \frac{m_i(m_i-1)}{\Lambda_0(C_i)^2\exp(2X_i\gamma_0)}\frac{\Lambda_0(C_i\wedge t)}{\Lambda_0(C_i)}\int_0^\tau \Delta_i(t)\frac{\frac{1}{n}\sum_j \Delta_j(t)\exp(X_j\gamma_0)X_j}{\frac{1}{n}\sum_j \Delta_j(t)\exp(X_j\gamma_0)}d\Lambda_0(t)n^{1/2}(\hat{\gamma}-\gamma_0) + o_p(n^{1/2}),\end{aligned}$$

which has mean zero and by Slutsky's lemma converges in distribution to a Normal.
It follows that $n^{1/2}Res(t;\hat{\eta},\hat{\gamma},\hat{\Lambda}_0,\hat{\Omega})$ converges in distribution to a Normal with mean zero.

B RESULTS: RESIDUAL DIAGNOSTICS

B.1 Residual diagnostics for the simulation study

We applied our residual diagnostic to three simulated datasets. The first had $\mu_0(t) = \log(1+t) + 2(t+1)$, the second took $\mu_0(t) = \sin(t) + 2(t+1)$ and the third used $\mu_0(t) = \sin(4t)$. As can be seen from Figure B1, there is no clear trend for the first two choices of μ_0, indicating no lack of fit, whereas the final plot shows distinct oscillations and an inadequate fit.

B.2 Residual diagnostics for the bladder cancer study

As can be seen from Figure B2, the residual diagnostics show no lack of fit in the chosen spline basis for the intercept function.

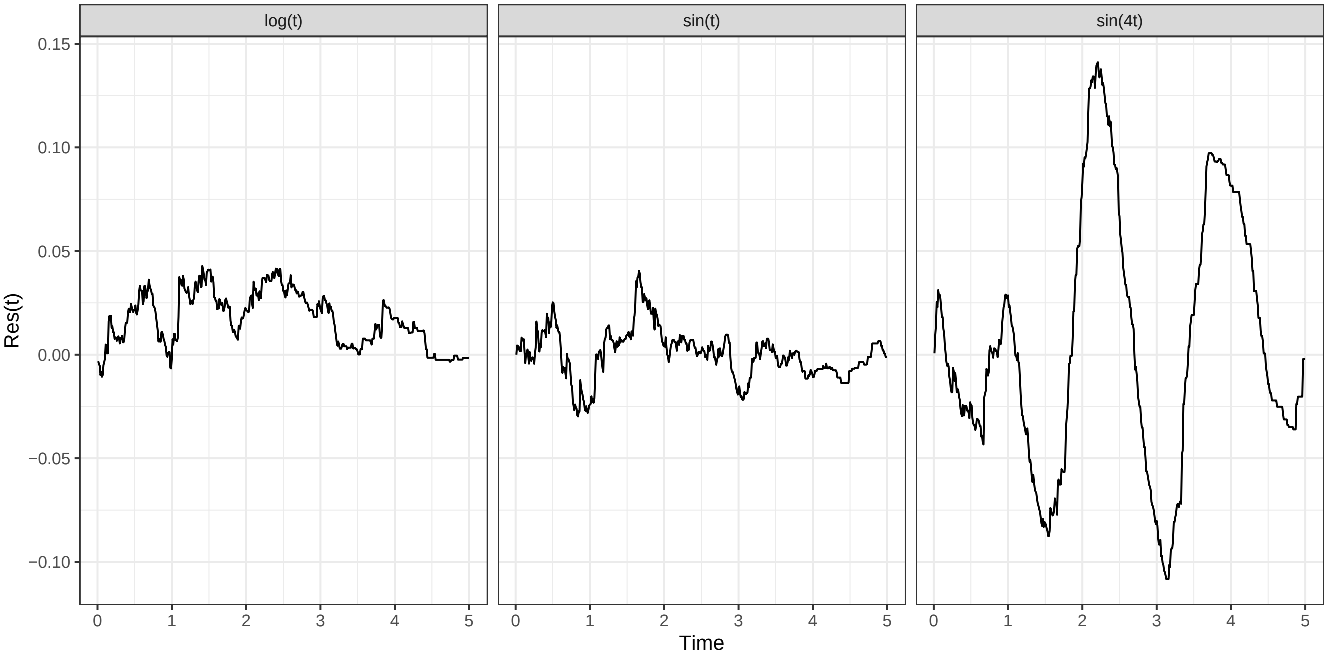

FIGURE B1 Residual diagnostics for three simulated datasets using $\mu_0(t) = \log(1+t)+2(t+1)$ (left), $\mu_0(t) = \sin(t)+2(t+1)$ (centre), and $\mu_0(t) = \sin(4t)$ (right).

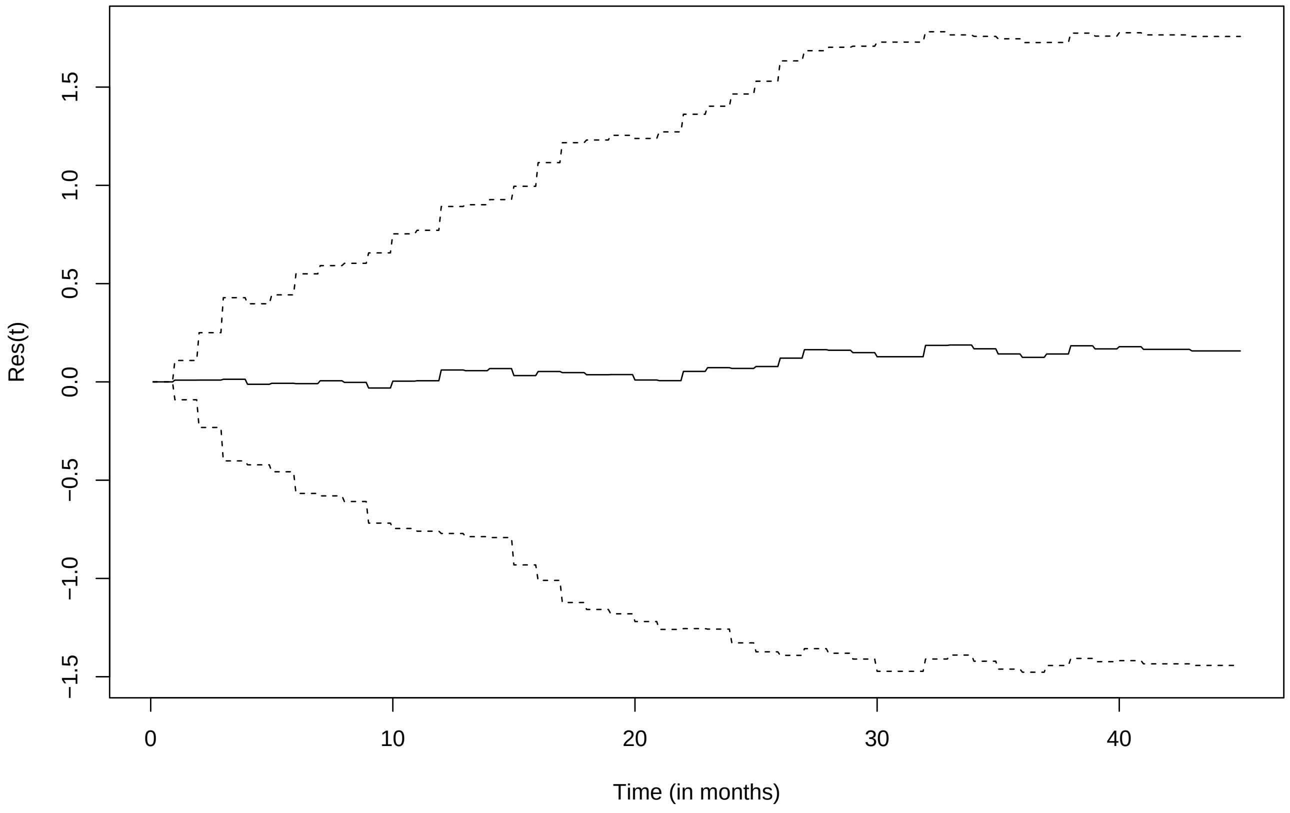

FIGURE B2 Residual diagnostics for the bladder cancer study, with 95% bootstrap confidence interval.

C SUPPLEMENTARY SIMULATION RESULTS: BOOTSTRAP COVERAGE

	n = 100	n = 200	n = 350	n = 500
Bias	0.00	0.01	0.01	0.00
Empirical Standard Error	0.11	0.08	0.06	0.05
Average Standard Error	0.11	0.08	0.06	0.05
Coverage Probability	0.95	0.94	0.95	0.95

TABLE C1 Bootstrap coverage probabilities for the extended Sun model corresponding to the base case in Table 2. Standard errors were estimated using 100 bootstrap replications. The average standard error is the median over the 1000 iterations.

D CODE USED FOR SIMULATION STUDY AND FIGURE GENERATION

The R Version 4.2.2[17] code has been uploaded to the Supplementary Material, which assumes that all files are located in the same workspace. See the README file for instructions.
