# Supplementary material for: Intercept Estimation of Semi‐Parametric Joint Models in the Context of Longitudinal Data Subject to Irregular Observations
Source: Biom J. 2025 Nov 6;67(6):e70088. doi: 10.1002/bimj.70088 (PMC12592789; doi:10.1002/bimj.70088)
Supplement: Supplementary file 2 — Supporting file 2: bimj70088‐sup‐0002‐DataCode.zip [file BIMJ-67-e70088-s002.zip › Code and Data/paper_figures/figure 1 - intercept_plot.pdf]

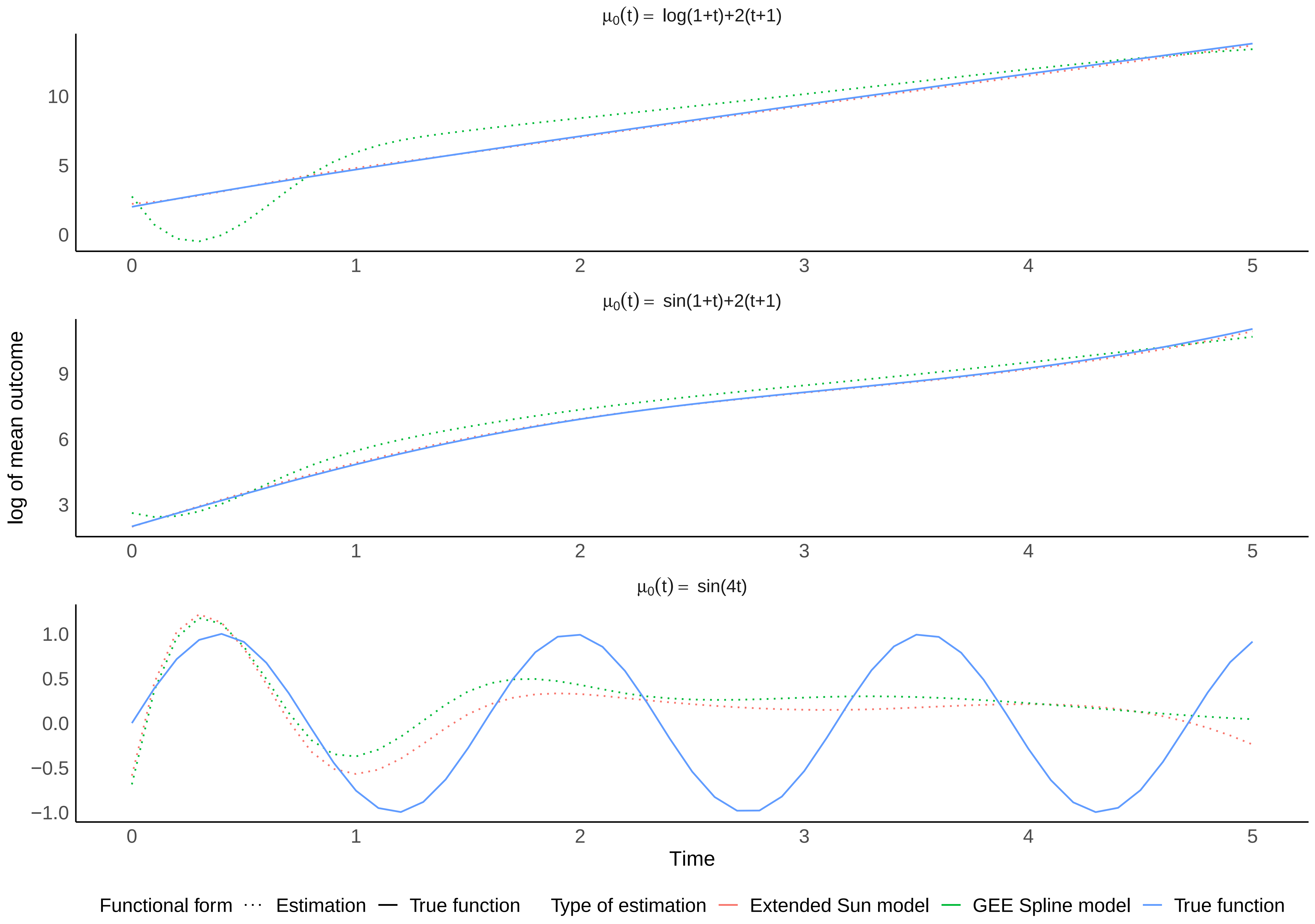

μ0(t) = log(1+t)+2(t+1)
μ0(t) = sin(1+t)+2(t+1)
μ0(t) = sin(4t)
log of mean outcome
Time
Functional form
Estimation
True function
Type of estimation
Extended Sun model
GEE Spline model
True function
