# Supplementary figures and images for: Intercept Estimation of Semi‐Parametric Joint Models in the Context of Longitudinal Data Subject to Irregular Observations
Source: Biom J. 2025 Nov 6;67(6):e70088. doi: 10.1002/bimj.70088 (PMC12592789; doi:10.1002/bimj.70088)

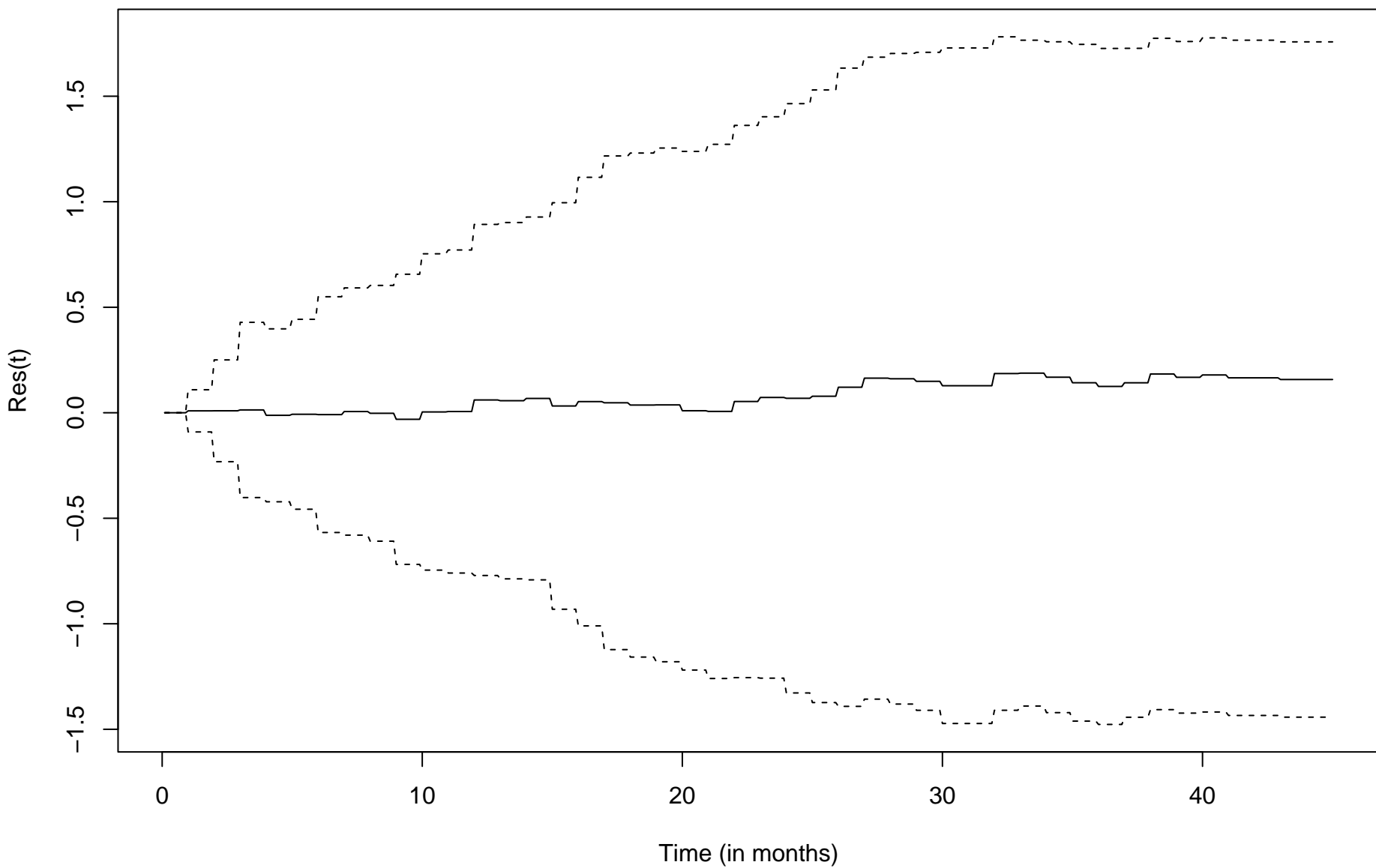

Supplement: Supplementary file 2 — Supporting file 2: bimj70088‐sup‐0002‐DataCode.zip [file BIMJ-67-e70088-s002.zip › Code and Data/paper_figures/figure b2 - Bladder_diagnosticplot.pdf]

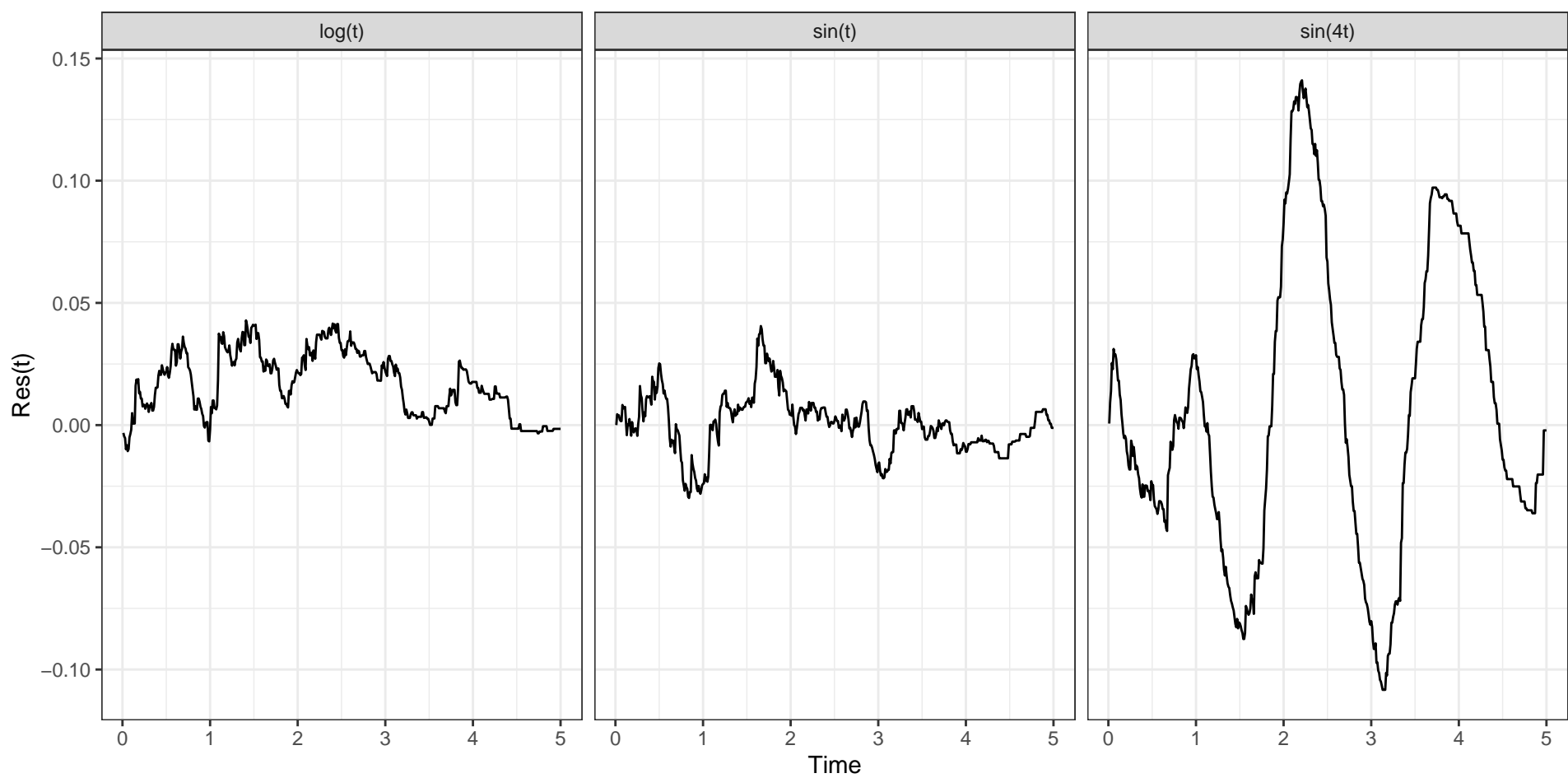

Supplement: Supplementary file 2 — Supporting file 2: bimj70088‐sup‐0002‐DataCode.zip [file BIMJ-67-e70088-s002.zip › Code and Data/paper_figures/figure b1 - SimulationResidualPlots.pdf]

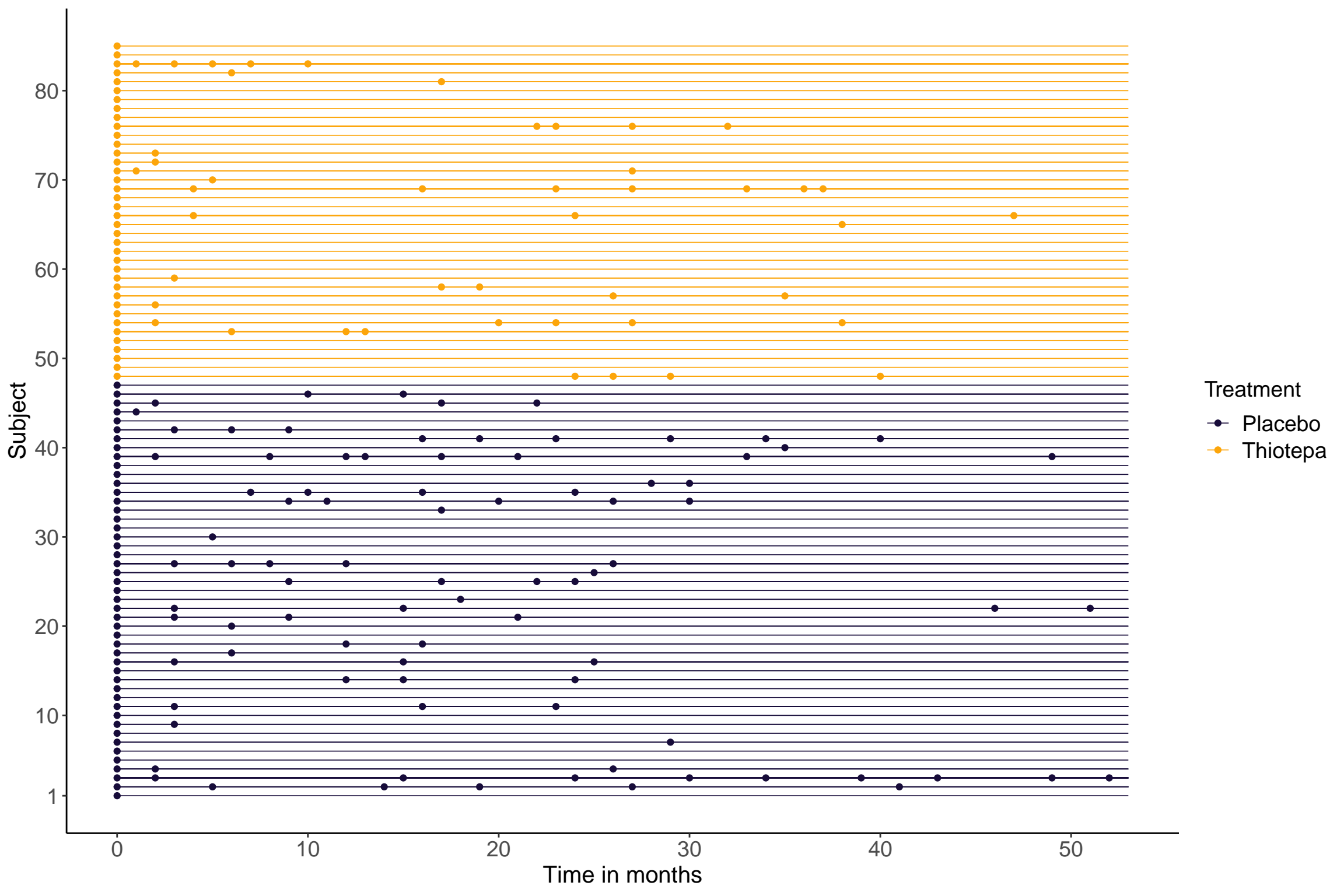

Supplement: Supplementary file 2 — Supporting file 2: bimj70088‐sup‐0002‐DataCode.zip [file BIMJ-67-e70088-s002.zip › Code and Data/paper_figures/figure 2 - abacus_plot.pdf]

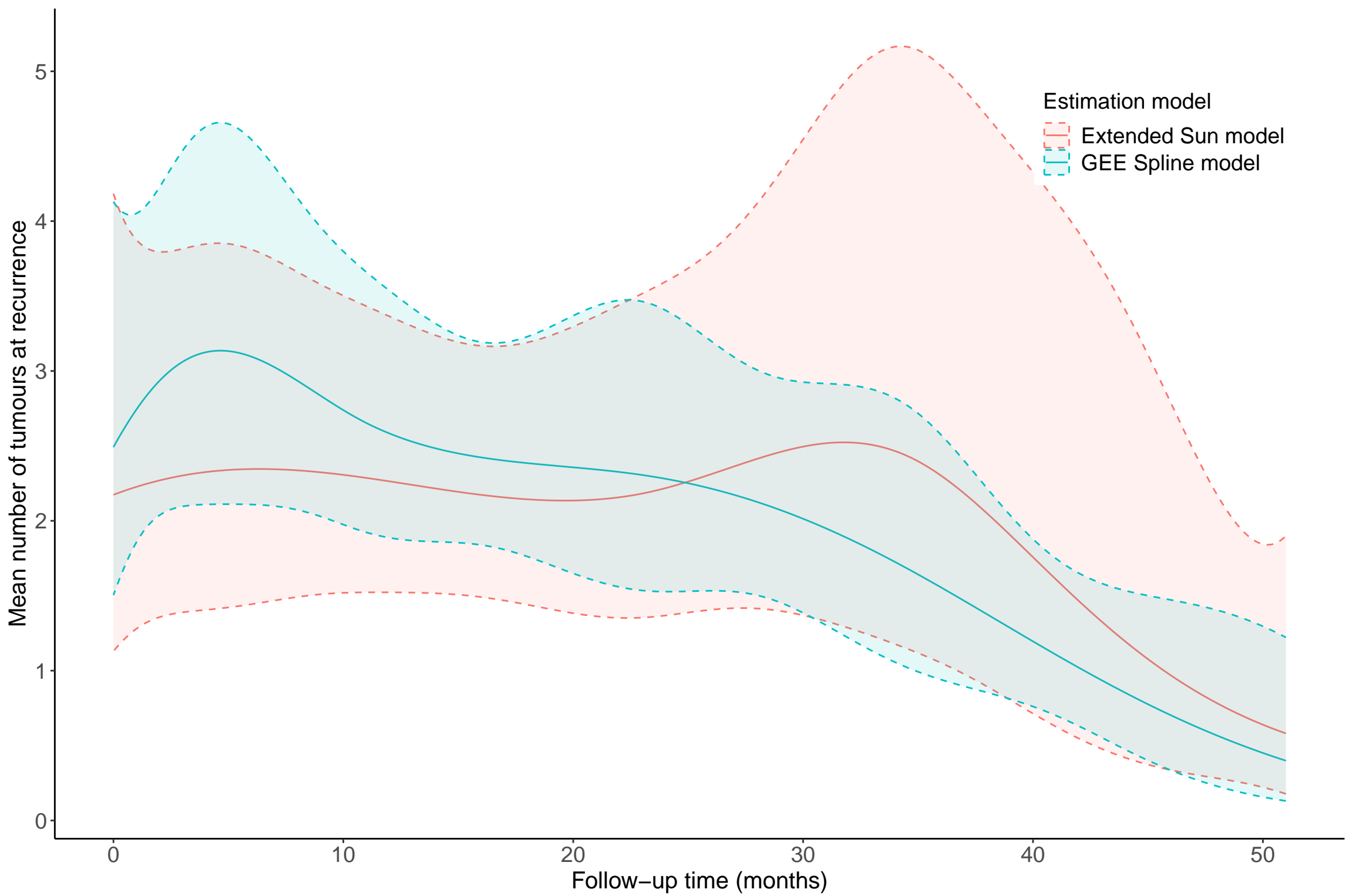

Supplement: Supplementary file 2 — Supporting file 2: bimj70088‐sup‐0002‐DataCode.zip [file BIMJ-67-e70088-s002.zip › Code and Data/paper_figures/figure 3 - dataanalysis_plot.pdf]
